# A self-powered multifunctional dressing for active infection prevention and accelerated wound healing

**DOI:** 10.1126/sciadv.adc8758

**Published:** 2023-01-25

**Authors:** Snigdha Roy Barman, Shuen-Wen Chan, Fu-Cheng Kao, Hsuan-Yu Ho, Imran Khan, Arnab Pal, Chih-Ching Huang, Zong-Hong Lin

**Affiliations:** ^1^ Institute of Biomedical Engineering, National Tsing Hua University, Hsinchu 30013, Taiwan.; ^2^ International Intercollegiate Ph.D. Program, National Tsing Hua University, Hsinchu 30013, Taiwan.; ^3^ Department of Biomedical Engineering and Environmental Sciences, National Tsing Hua University, Hsinchu 30013, Taiwan.; ^4^ Department of Orthopaedic Surgery, Spine Section, Chang Gung Memorial Hospital, Taoyuan 33305, Taiwan.; ^5^ College of Medicine, Chang Gung University, Taoyuan 33302, Taiwan.; ^6^ Department of Power Mechanical Engineering, National Tsing Hua University, Hsinchu 30013, Taiwan.; ^7^ Institute of NanoEngineering and Microsystems, National Tsing Hua University, Hsinchu 30013, Taiwan.; ^8^ Department of Bioscience and Biotechnology, National Taiwan Ocean University, Keelung 202301, Taiwan.; ^9^ Center of Excellence for the Oceans, National Taiwan Ocean University, Keelung 202301, Taiwan.; ^10^ School of Pharmacy, College of Pharmacy, Kaohsiung Medical University, Kaohsiung 80708, Taiwan.; ^11^ Department of Chemistry, National Tsing Hua University, Hsinchu 30013, Taiwan.; ^12^ Frontier Research Center on Fundamental and Applied Sciences of Matters, National Tsing Hua University, Hsinchu 30013, Taiwan.; ^13^ Department of Biomedical Engineering, National Taiwan University, Taipei 10617, Taiwan.

## Abstract

Interruption of the wound healing process due to pathogenic infection remains a major health care challenge. The existing methods for wound management require power sources that hinder their utilization outside of clinical settings. Here, a next generation of wearable self-powered wound dressing is developed, which can be activated by diverse stimuli from the patient’s body and provide on-demand treatment for both normal and infected wounds. The highly tunable dressing is composed of thermocatalytic bismuth telluride nanoplates (Bi_2_Te_3_ NPs) functionalized onto carbon fiber fabric electrodes and triggered by the surrounding temperature difference to controllably generate hydrogen peroxide to effectively inhibit bacterial growth at the wound site. The integrated electrodes are connected to a wearable triboelectric nanogenerator (TENG) to provide electrical stimulation for accelerated wound closure by enhancing cellular proliferation, migration, and angiogenesis. The reported self-powered dressing holds great potential in facilitating personalized and user-friendly wound care with improved healing outcomes.

## Teaser

Thermocatalytic generation of H2O2 by Bi2Te3 NPs and electrical stimulation from TENG can improve wound healing.

## INTRODUCTION

With the advancement of the Internet of Things (IoT), the world has witnessed an ever-growing concept of point of care, which is defined by a more personalized and minimally invasive approach to health care with better health outcomes and higher patient convenience (*1*–*3*). The point-of-care paradigm usually comprises wearable medical devices integrated with flexible electronics to accomplish conformal contact with skin and has emerged as a unique solution for the monitoring and on-demand treatment of diseases such as chronic wounds (*4*–*7*). Recent advances in medical technology have yielded advanced treatment strategies for chronic wounds, such as hyperbaric oxygen therapy (*8*), ultrasound (*9*), electromagnetic therapy (*10*, *11*), negative pressure therapy (*12*, *13*), photothermal therapy (*14*, *15*), and electrical stimulation (ES) (*16*, *17*). In particular, ES has emerged as an indispensable tool for wound care owing to its noninvasive nature, minimal side effects, and simplicity of operation (*16*, *18*). Current modalities of therapeutic ES include high-voltage pulsed current (HVPC), low-intensity DC (LIDC), and high-frequency AC electric fields (ACEF), which require bulky extracorporeal devices and professional operators, necessitating patient hospitalization (*6*, *19*, *21*). Furthermore, such devices rely on an external power source, which restricts their capabilities because of the rigid structure, limited lifetime, and risks for environmental pollution (*4*, *21*). Owing to these challenges, it is becoming increasingly important to develop wearable and miniaturized self-powered devices for wound healing that can render a comfortable experience for the patients while being treated.

Recently, triboelectric nanogenerators (TENGs) have emerged as promising self-powered platforms because of their compelling characteristics, such as light weight, flexibility, conformability, easy fabrication, and high energy conversion efficiency (*22*–*24*). TENGs can transform biomechanical energy from human movements into electricity, which has been used for myriad biomedical applications as a power source for medical devices (*25*–*27*), and sensors for monitoring physiological parameters, including heart rate (*28*, *29*), respiration rate (*30*, *31*), pulse (*32*), strain (*33*), and pressure (*34*). By further integrating stretchable electrodes such as laser-induced graphene foams (*34*, *35*), thermoplastic polyurethane nanofibers (*36*), and so on with TENGs, highly stretchable self-powered platforms can be developed, which can generate electricity by maintaining a conformal contact with the skin (*37*). These recent developments has stirred a lot of interest in using the TENGs as an on-body ES module for delivering therapeutic electrical impulses, particularly for wound healing (*16*, *20*, *21*) and bone regeneration (*38*). Despite encouraging developments, providing effective ES via TENG is often challenged in terms of the biocompatibility, wearability, conformability, durability, and inadequate functionalities (*39*–*42*). The intensity of the EF generated by wearable TENGs is not sufficient to heal complicated chronic wounds that are often infected with pathogenic bacteria.

Bacterial growth in wound tissue is the most critical factor contributing to delayed healing due to the secretion of virulent enzymes, which destroys the host tissue and disrupts wound recovery (*43*, *44*). Therefore, it is crucial to develop synergistic treatment approaches into a single platform that can simultaneously impede bacterial growth and heal chronic wounds for timely patient recovery. Recently, several antibacterial strategies have been integrated with wound repair approaches that use the in situ generation of reactive oxygen species (ROS), such as hydrogen peroxide (H_2_O_2_), hydroxyl radicals (·OH), and superoxide (·O_2_^−^) to produce oxidative stress and bacterial cell damage (*45*, *46*). Overall, H_2_O_2_ is less reactive and more stable than other ROS (*47*). H_2_O_2_ can be generated by different chemical methods, such as anthraquinone-catalyzed *(48) *and electrocatalytic reduction of oxygen or by physical methods, such as plasma (*49*), photocatalysis (*50*), and piezocatalysis (*51*). Photocatalysts and piezocatalysts have been widely explored to generate H_2_O_2_; however, their round-the-clock performance is hindered because of the unavailability of light and suitable mechanical forces in the environment (*52*), thereby limiting their application in clinical scenarios. On the other hand, temperature is one of the most ubiquitous factors in our daily life, and temperature differences exist almost everywhere in our surrounding environment. Recently, the catalytic activity of thermoelectric materials, termed thermocatalysts, has been reported for H_2_O_2_ generation owing to their temperature difference–induced electron-hole pair separation, paving the way for the development of excellent alternatives to traditional photocatalysts and piezocatalysts (*52*). By using the naturally existing temperature difference in the bacteria-infected wound bed, thermocatalysts can spontaneously produce H_2_O_2_ in situ, which can immensely contribute to combating bacteria at the wound site for expedited wound healing.

Here, we report the development of a wearable self-powered wound dressing that can be triggered by diverse stimuli from the patient’s body (mechanical motions and temperature gradient) and can provide on-demand treatment for both normal and infected wounds. The tailored dressing was fabricated by integrating a thermocatalyst and TENG into a single system, where the thermocatalyst controllably produces H_2_O_2_ for in situ bacterial eradication, while the TENG locally generates ES to promote wound healing. The near–room temperature thermoelectric material bismuth telluride (Bi_2_Te_3_) was used as a thermocatalyst owing to its high Seebeck coefficient and excellent physical properties (*53*, *54*). Under different temperature gradients, a mild amount (≤10 μM) of H_2_O_2_ is generated by Bi_2_Te_3_ nanoplates (Bi_2_Te_3_ NPs), which will not trigger any adverse effect in normal tissues but at the same time is highly effective for antibacterial function. The amount of H_2_O_2_ produced by the thermocatalyst is controllable, resulting in antibacterial activity that can be easily modulated depending on the severity of the infection at the wound site. Owing to its multifunctional capabilities, a tunable treatment pathway of the as-prepared wound dressing regulated by the wound type was demonstrated in an in vivo animal model. In the case of normal skin wounds, the wound dressing was connected to an arch-shaped TENG (a-TENG) to generate pulsed EFs to accelerate wound recovery, whereas EF from the a-TENG were synergistically applied with Bi_2_Te_3_ NPs activated under a temperature difference to inhibit bacteria and promote subsequent healing of the wound for effective treatment of infected wounds. The concept and results presented in this work provide an advanced and power-free strategy for repairing versatile wounds.

## RESULTS

### Design of the self-powered wound dressing

**Fig. 1.**
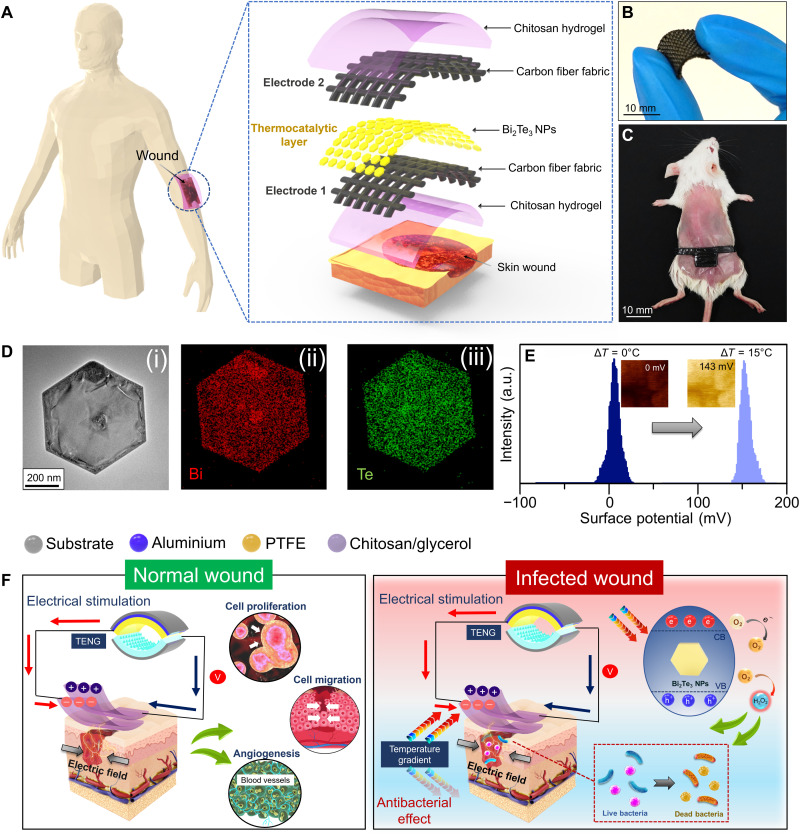
Schematic representation of a multifunctional self-powered wound dressing and its working mechanism. (**A**) Structural design of the wound dressing with Bi_2_Te_3_ NPs as the thermocatalytic layer, chitosan hydrogel as the encapsulation layer, and CFF as the electrodes. (**B**) Flexible nature of the wound dressing. (**C**) Implementation of the wound dressing on a full-thickness wound created on the dorsal region of mice. (**D**) HRTEM image and elemental mapping images of Bi_2_Te_3_ NPs. (**E**) Change in the surface potential of Bi_2_Te_3_ NPs probed by KPFM at different temperature gradients. (**F**) Mechanism of the wound dressing for healing normal and infected wounds. a.u., arbitrary units.

A multifunctional wound dressing responsive to temperature gradients and mechanical stimuli is proposed as an appealing strategy for personalized wound treatment. The layer-by-layer approach was used to design the multifunctional dressing, which typically comprised a thermocatalytic layer (i.e., Bi_2_Te_3_ NPs) and a mechanical energy harvesting layer [i.e., chitosan coated carbon fiber fabrics (CFFs)] (Fig. 1A). The layered dressing structure is mainly composed of two CFF-based electrodes (top and bottom) coated with chitosan hydrogel on the exterior side. Chitosan hydrogel was used as a triboelectric and encapsulation layer because of its excellent biocompatibility, bioabsorbability, and biodegradability. The developed dressing is highly flexible (Fig. 1B), thus offering substantial conformal contact with full-thickness wounds (1 cm by 1 cm^2^) created on the dorsal region of mice (Fig. 1C). The wound dressing was integrated by vertically assembling two chitosan/CFFs, and two strips of uncoated CFFs from each layer placed outside the wound area functioned as electrodes that were connected to the TENG for ES. The inner side of the bottom CFF electrode was coated with thermocatalytic Bi_2_Te_3_ NPs, which can be triggered by a temperature gradient. In our previous study, Bi_2_Te_3_ NPs generated electron-hole pairs in response to an applied temperature gradient to effectively catalyze the reduction of molecular oxygen into ROS, such as H_2_O_2_. The high-resolution transmission electron microscopy (HRTEM) image in Fig. 1D reveals that the Bi_2_Te_3_ material forms a hexagonal NP-like morphology. In addition, well-defined lattice fringes with an interatomic spacing of 0.22 nm corresponding to the (110) planes of Bi_2_Te_3_ were observed, as shown in fig. S1 (A and B). Moreover, a homogeneous distribution of Bi and Te in a single NP was observed from the elemental mapping results obtained from energy-dispersive x-ray spectroscopy (EDX) analysis (Fig. 1D). Furthermore, the X-ray diffraction (XRD) patterns shown in fig. S1C confirmed the highly crystalline nature of the as-formed Bi_2_Te_3_ NPs.

To evaluate the voltage generation in thermoelectric materials in the presence of a temperature difference, surface potential analysis was carried out by Kelvin probe force microscopy (KPFM) integrated with a thermal stage (Fig. 1E and fig. S2). In the absence of a temperature gradient (Δ*T*), i.e., Δ*T* = 0°C, Bi_2_Te_3_ NPs showed negligible surface potential (⁓0 mV); however, when Δ*T* was increased to 7° and 15°C, the surface potential values of Bi_2_Te_3_ NPs were markedly enhanced to 43 and 137 mV, respectively (fig. S2). A slightly lower surface potential value (121 mV) was recorded under negative temperature gradients (Δ*T* = −15°C), suggesting that voltage generation depends not only on the applied temperature difference but also on the varied Seebeck coefficient within different temperature ranges. When the Bi_2_Te_3_ NPs were subjected to a temperature gradient, the potential difference between the KPFM tip and sample increased compared to the thermal equilibrium condition due to the increased surface charges of Bi_2_Te_3_ NPs, ultimately leading to a decrease in the work function of the material. A field-emission scanning electron microscopy (FESEM) image of the chitosan layer revealed the porous structure of the wound dressing (fig. S3A). The elemental mapping showed a uniform spatial distribution of C and O in the chitosan layer (fig. S3, B and C). Moreover, the adhesiveness of the wound dressing was evaluated at varying relative humidity (RH) conditions using the standard lap-shear test (fig. S4A). A negligible decrease in the adhesive strength was observed at high RH of 70%, indicating that the as-fabricated wound dressing can maintain adequate contact with the wound tissue even in the presence of sweat (fig. S4B). The as-prepared self-powered wound dressing can also be fabricated into different morphologies to fit variable wound shapes, including the irregular ones, and can be easily adapted to distinct wound sites, which validates the highly customizable nature of the as-fabricated dressing (fig. S5, A and B). Subsequently, the self-powered wound dressing was used as a wearable device to treat both normal and infected wounds (Fig. 1F). Two on-demand treatment strategies have been demonstrated on the basis of the wound type. The electrical output from the TENG activates the wound dressing to accelerate the healing process, while the presence of a temperature gradient effectively catalyzes the production of H_2_O_2_ to eradicate bacteria from the wound site.

### Antibacterial performance of thermocatalytic Bi_2_Te_3_ NPs

**Fig. 2.**
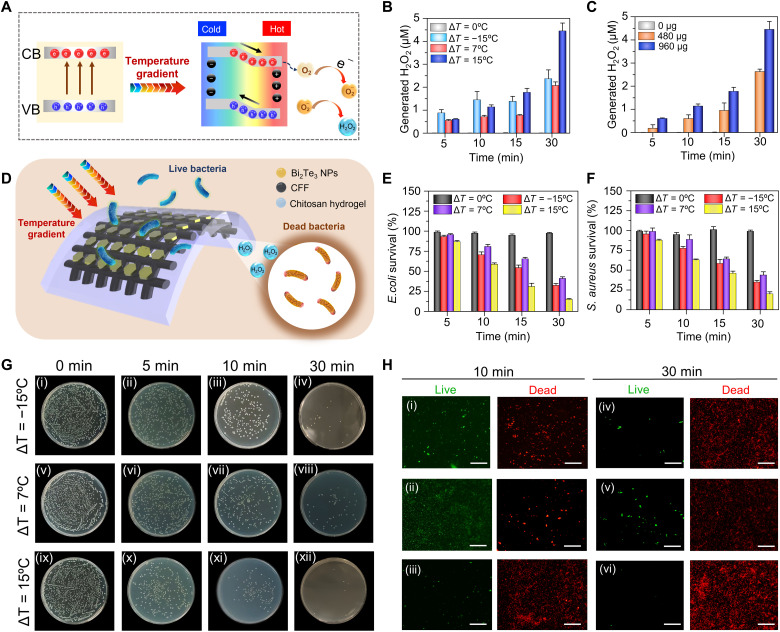
Temperature-dependent antibacterial performance of Bi_2_Te_3_ NPs. (**A**) Schematic illustration of the thermocatalytic mechanism for H_2_O_2_ generation under a temperature gradient created because of the applied temperature difference. (**B**) Generation of H_2_O_2_ by Bi_2_Te_3_ NPs under different temperature gradients at different time intervals. (**C**) Generation of H_2_O_2_ by different amounts of Bi_2_Te_3_ NPs at different time intervals at Δ*T* = 15°C. (**D**) Schematic representation of the antibacterial mechanism of Bi_2_Te_3_ NPs. (**E** and **F**) Antibacterial efficiency of Bi_2_Te_3_ NPs for *Escherichia coli* [*E. coli*; Gram-negative bacterium (E)] and *Staphylococcus aureus* [*S. aureus*; Gram-positive bacterium (F)] at various temperature gradients. (**G**) Plating results showing the concentration of *E. coli* after treatment at different temperature gradients. (**H**) Images of live (green fluorescence) and dead (red fluorescence) bacterial cells following different temperature gradients after 10 and 30 min of treatment. Scale bars, 100 μm. Results are plotted as means ± SD (*n* = 3).

It has been reported that thermoelectric materials can efficiently facilitate electron-hole pair separation in response to a temperature gradient (*52*). The negative charge migrates from the hot side to the cold side of the Bi_2_Te_3_ NP, thus generating a potential difference between the two ends (Fig. 2A). As a result, both the valence and conduction bands bend across the material to catalyze the formation of ·O_2_^−^ radicals from molecular oxygen. Subsequently, electrons from the conduction band of Bi_2_Te_3_ NP migrate to the solution to produce H_2_O_2_ (·O_2_^−^ + e^−^ + 2H^+^ → H_2_O_2_). Therefore, thermocatalytic generation of H_2_O_2_ from Bi_2_Te_3_ NP–coated wound dressings was evaluated at different temperature gradients (Fig. 2B). Obvious amounts of H_2_O_2_ (>0.5 μM) were generated under various thermal gradients (Δ*T* = −15°, 7°, and 15°C) even after 5 min of treatment. The results showed the time-dependent formation of H_2_O_2_. Expectedly, maximum H_2_O_2_ was generated at a positive temperature gradient (Δ*T* = 15°C, 4.4 μM), which is ⁓2.2-fold higher than that at −15°C, mainly due to an abrupt increase in the surface potential, as seen in fig. S2. The negligible amount of H_2_O_2_ in the control (Δ*T* = 0°C) groups confirm that the catalytic reaction is governed only by the applied temperature difference (fig. S6A). Moreover, fig. S6B shows that the H_2_O_2_ generation efficiency of Bi_2_Te_3_ NP–coated dressings is 4.3-fold higher than that of uncoated dressings. Dose-dependent investigation revealed that the production of H_2_O_2_ was further improved as the amount of Bi_2_Te_3_ NPs increased (Fig. 2C and fig. S7). In addition, negligible effect of RH on the H_2_O_2_ generation was observed, which revealed the robust nature of the Bi_2_Te_3_ NP–functionalized wound dressings against humidity changes in real environments (fig. S8, A and B). Nevertheless, the best catalytic performance of Bi_2_Te_3_ NPs for H_2_O_2_ generation was obtained with a higher catalytic amount at an elevated temperature gradient. Owing to the ROS-generating capabilities, thermocatalytic Bi_2_Te_3_ NP–functionalized wound dressings were further used to inactivate bacteria and carry out self-powered disinfection (Fig. 2D). The antibacterial performance of the wound dressing was assessed against *Escherichia coli (E. coli)* and *Staphylococcus aureus (S. aureus)* through a standard plate-counting method and live/dead bacterial assays. The agar plating images and their respective quantitative results showed that as the temperature gradient increased from 0° to 15°C, the survival rates of *E. coli* substantially decreased from 100 to 16%, respectively (Fig. 2, E and F). Similarly, applied temperature gradient–dependent inhibition of bacterial growth was demonstrated against *S. aureus* (Fig. 2G and fig. S9). SYTO 9/propidium iodide (PI) staining of *E. coli* was performed to evaluate the integrity of the bacterial cell membrane. *E. coli* suspension treated under different temperature gradients indicated a large number of dead bacteria only after 10 min, which further increased after 30 min (Fig. 2H). These results imply a direct relationship between the amount of H_2_O_2_ generated and the antibacterial performance. The superior bacteriostatic activity is attributed to the enormous amount of H_2_O_2_ produced by Bi_2_Te_3_ NPs at a higher temperature gradient. Furthermore, we demonstrated that the bacterial viability of *E. coli* and *S. aureus* at Δ*T* = 15°C was dependent on the Bi_2_Te_3_ NP loading concentration (fig. S10). The amount of H_2_O_2_ released from the dressing could be easily controlled by the external temperature gradient, Bi_2_Te_3_ NP loading concentration, and treatment time; thus, wound dressings were used for further in vivo studies to examine the potential of self-powered antimicrobial systems.

### Design and output performance of the TENG

**Fig. 3.**
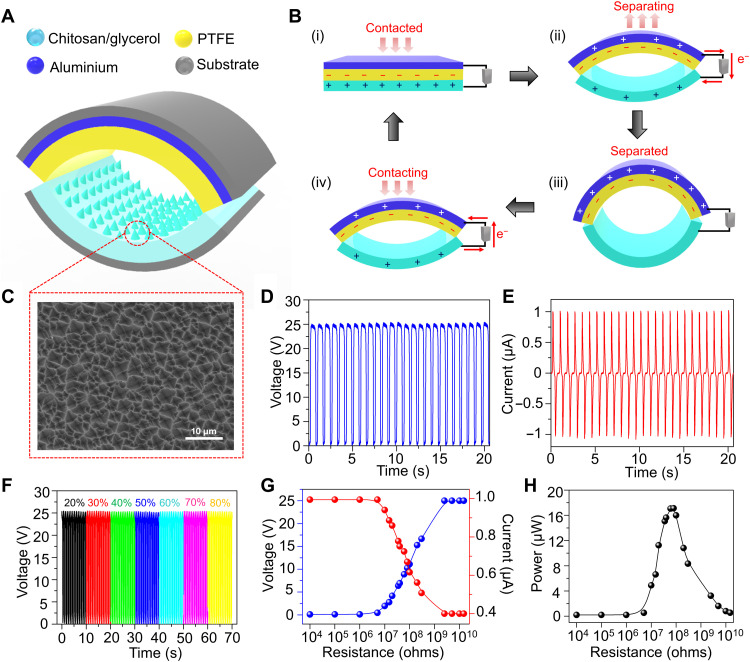
Fabrication and output performance of the a-TENG. (**A**) Schematic showing the structure of the a-TENG. (**B**) Working mechanism of the a-TENG under an external force. (**C**) FESEM image of the chitosan/glycerol layer of the a-TENG. (**D** and **E**) Output voltage (D) and current (E) of the a-TENG when driven by a linear motor. (**F**) Output voltage of the a-TENG at different RHs. The output voltage of the a-TENG remains the same even at a higher RH. (**G**) Dependence of the voltage and current of the TENG under various external load resistances. (**H**) Output power generated by the a-TENG under different external load resistances.

Traditional plate-based TENGs are not able to achieve full contact separation when an external force is applied because of rigidity in triboelectric layers. To address this limitation, an arch-shaped (a-TENG) with dimensions of 2 cm by 2 cm operating in vertical contact-separation mode was fabricated using chitosan/glycerol film and polytetrafluoroethylene (PTFE) as triboelectric layers (Fig. 3A). While the chitosan/glycerol film also functioned as the electrode layer, aluminum was deposited on the back side of PTFE, which served as the conductive layer. Moreover, polyethylene terephthalate (PET) was used as the substrate for the design of the a-TENG. Owing to its simple fabrication process, flexibility, and durability, the as-designed a-TENG is suitable for in vivo applications. The working mechanism of the a-TENG is illustrated in Fig. 3B. Initially, the arch shape does not allow any contact between triboelectric layers; however, upon application of external force, triboelectric layers come in contact with each other, resulting in charge transfer between the two layers. Depending on their electron-donating ability, electrons are transferred from the chitosan/glycerol film to PTFE, resulting in positive and negative charges on the surface of the chitosan/glycerol film and PTFE, respectively. However, because of the balanced net charge, electron flow does not occur in the external circuit (Fig. 3B, i). Once the externally applied force is withdrawn, the triboelectric layers revert to their native arch-like structure, which then disrupts the electrical equilibrium and drives the electrons in the external circuit (Fig. 3B, ii). This process of electron flow continues until a new equilibrium state is achieved, and the two triboelectric layers are completely separated (Fig. 3B, iii). When the force is applied again, a negative potential difference is generated that facilitates electron flow in the opposite direction to achieve electrical neutrality (Fig. 3B, iv). Moreover, microstructures were patterned on the surface of the chitosan/glycerol film via the replica molding method to enhance the effective contact area, which boosted the triboelectric output (Fig. 3C). Furthermore, the output characteristics of the as-fabricated TENG were evaluated by using a linear motor operating at a frequency of 1 Hz to obtain a stable mechanical force. As shown in Fig. 3 (D and E), the output voltage and current of the as-fabricated a-TENG reached 25 V and 1 μA, respectively. The output characteristics of the a-TENG remained unaltered over a wide range of environmental humidity and temperature changes, which is crucial for in vivo studies (Fig. 3F and fig. S11). In addition, the output voltage and current under different external resistances from 10 kilohms to 10 gigohms were tested, as shown in Fig. 3G. The corresponding power output is displayed in Fig. 3H, which shows that the maximum power of 18 μW is achieved at 60 megohm. Furthermore, the wearability of the a-TENG was demonstrated by wrapping the device around the dorsal region of mice (fig. S12A and movie S1). In the resting state, the arch shape of the TENG remains intact; however, in the active state (walking or running), the dorsal region becomes elevated, allowing the chitosan/glycerol layer to contact PTFE to generate the triboelectric signal (fig. S12B). Regular discrete voltage peaks were observed with every step of mouse motion, verifying that continuous ES can be achieved through such motion (fig. S12C). In the calm state, no notable voltage signal was generated; however, as the mice started to walk, a voltage output of 6.5 V was detected, which was further increased in the running state (25 V), confirming the strong correlation between TENG performance and the motion of mice.

### Effect of TENG-induced ES on cellular behavior

**Fig. 4.**
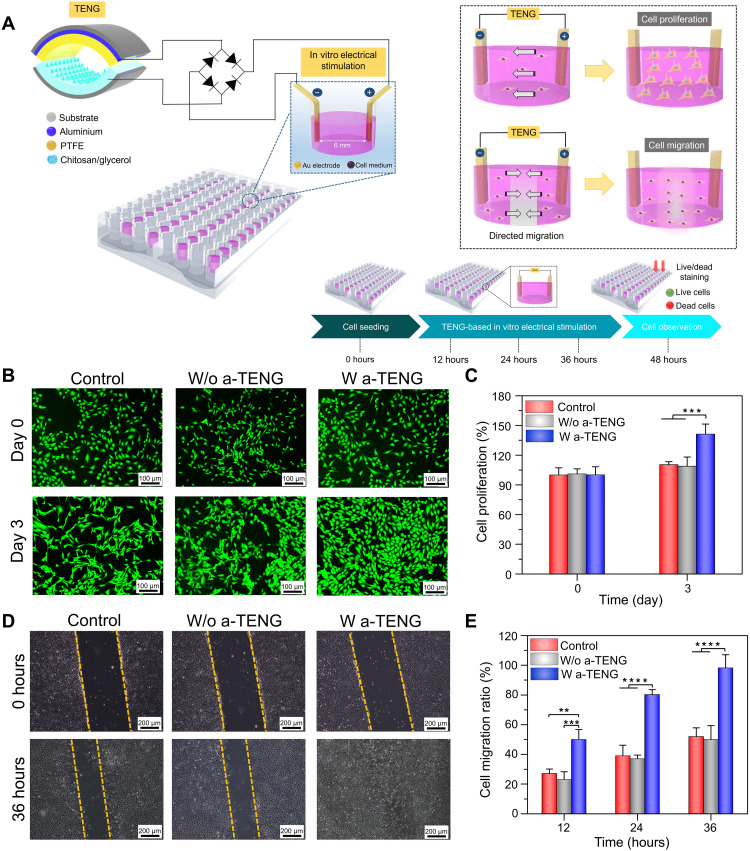
In vitro cellular responses of fibroblasts subjected to ES from the a-TENG. (**A**) Schematic representation showing the influence of ES on cell proliferation and migration. (**B**) Fluorescence images of fibroblasts after ES from a-TENG recorded on days 0 and 3. (**C**) Cell proliferation of fibroblasts after 3 days of a-TENG–based ES. (**D**) Cell migration images of fibroblasts after 36 hours of ES from the a-TENG. (**E**) Corresponding cell migration ratio of fibroblasts under ES compared to the control groups. Results are plotted as means ± SD (*n* = 4); *P < 0.05, **P < 0.01, ***P < 0.001, and ****P < 0.0001.

Fibroblasts play vital roles in the wound healing process and are usually involved in the migration, proliferation, and degradation of fibrin clots and the production of new extracellular matrix (ECM) components and several cytokines. The influence of electrical stimuli from a-TENG on fibroblast cells was studied in vitro by examining cell proliferation and migration. In a typical experimental setup, the a-TENG was connected to a rectifier to transform the AC into DC signal, generating an output voltage of 25 V at a constant frequency of 1 Hz. It has been reported that compared to AC, DC-based electrical output assists in directional migration and enhances the rate of angiogenesis, which, in turn accelerates the wound healing process (*55*, *56*). The poor performance of AC-induced EFs is probably attributed to the lack of polarity in the opposing terminals (*57*). As shown in Fig. 4A, a pair of Au foil electrodes was placed in a well of the cell plate in a parallel orientation and connected to the a-TENG. Subsequently, the effects of various parameters such as treatment time, distance between the electrodes, and type of electrode on cell proliferation were systematically investigated. Figure S13A shows a gradual increase in the cell proliferation rate, reaching 42% in 30 min, which decreased instantly upon further stimulation because of cell damage caused by the Joule healing effect, resulting from prolonged ES durations (*58*). An increase in the duration of ES shifted the optimal EF strength required for promoting cell proliferation to lower values. In contrast, no obvious change was recorded for the control and without a-TENG (w/o a-TENG) groups (fig. S13B). The strength of the EF determines the fate of cell death, which can be greatly influenced by adjusting the distance between two Au electrodes placed in each cell well. The cell proliferation rate was boosted up to 141.2% as the interelectrode distance increased to 6 mm (fig. S13C). However, at a reduced interelectrode distance, increased EF strength leads to a change in the local pH of the medium from neutral to acidic, causing denaturation of proteins and alternation of normal cell functions (*59*). As a result, cell proliferation is decreased with decreasing distance between the two electrodes, as shown in fig. S13D. The rate of cell proliferation was markedly decreased (⁓120%) by using an Au wire–based electrode system instead of Au foil at a constant interelectrode distance of 6 mm, likely due to the relatively smaller surface area of the wire electrodes. A larger surface area of the foil electrodes enhances the charge storage capacity, thereby injecting a greater number of charges into the cell medium during ES, which results in a superior proliferation effect. Under optimal conditions, fibroblast cells electrically stimulated by the TENG for three consecutive days were labeled with Calcein-AM for observation under a fluorescence microscope (Fig. 4B). In contrast to the control and w/o TENG groups, stimulated fibroblast cells showed a 41.2% increase in cellular growth (Fig. 4C). Pulsed ES induces fibroblast proliferation by activating the transforming growth factor–β1–extracellular signal–regulated kinase (TGFβ1-ERK) pathway, which up-regulates several growth factors and cytokines, such as fibroblast growth factor 2 (FGF2), Delta-like non-canonical Notch ligand 1 (Dlk1), and so on (*60*). The results also suggested negligible cytotoxicity of Au electrodes used for proliferating the fibroblasts for up to 3 days. In addition, the developed TENG boosted the migration of fibroblast cells within 36 hours in the direction of the applied EF, unlike comparative groups, which migrated in arbitrary orientations (Fig. 4D). Directional migration enables contraction force toward the scratch area, leading to wound closure at a faster rate. Relative to those of the control and w/o TENG groups, stimulated cells migrated twofold faster (Fig. 4E). Last, the in vitro cytotoxicity of the Bi_2_Te_3_ NP–coated wound dressings was evaluated after coincubation with fibroblast cells for 24 hours (fig. S14).

### TENG-driven treatment strategy for healing normal wounds

**Fig. 5.**
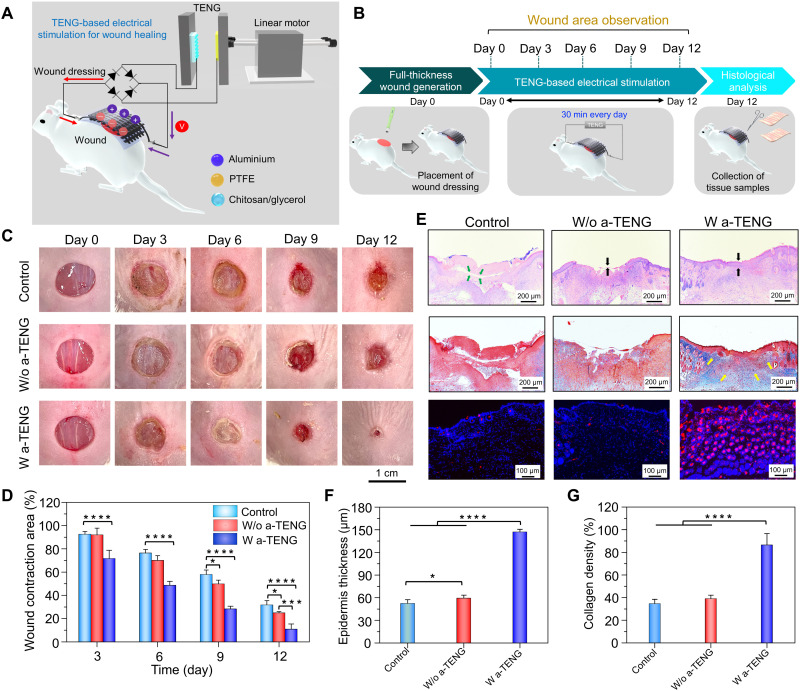
Effect of ES from the a-TENG on the healing of normal full-thickness wounds. (**A**) Schematic representation of wound dressing stimulated by the electrical output from a-TENG. (**B**) Schematic diagram of the experimental process throughout the 12-day period. (**C** and **D**) Digital photographs (C) and corresponding wound contraction area (D) of wounds taken on days 0, 3, 6, 9, and 12 for the control, w/o a-TENG, and w/ a-TENG groups. (**E**) H&E, Masson’s trichrome, and immunofluorescence staining for CD31 in wounds on day 12 following various treatments. (**F** and **G**) Quantitative results concerning the thickness of the epidermis (F) and collagen density in the dermal layer (G) of wounds on day 12 following various treatments. Results are plotted as means ± SD (*n* = 6); *P < 0.05, **P < 0.01, ***P < 0.001, and ****P < 0.0001.

To demonstrate practicality, the wearable self-powered dressing was directly applied for in vivo wound healing applications. A full-thickness circular wound with a diameter of 8 mm was created on the dorsal region of the mice. The dressing was positioned on top of the wound area with two fabric electrodes placed on each side of the wound such that the generated EF was perpendicular to the wound direction (Fig. 5A). The electrodes incorporated within the dressing was connected to the two terminals of the TENG after passing through a rectifier bridge, which generated a DC EF that could penetrate the dermis and intensify the endogenous EF to accelerate wound healing. During the ES study, TENG was controlled by the linear motor to produce a consistent mechanical force that generated an output voltage of 25 V at 1 Hz, which was conveyed to the wound dressing using conductive wires (Fig. 5B and fig. S15). To monitor the healing process, photographs of the wound area were captured at different time intervals over the course of 12 days (Fig. 5C). In the presence of an EF generated by the TENG, the wound area progressively contracted to 71.59% along the EF direction, compared to that of the control (92.4%) and w/o a-TENG (91.9%) groups, on day 3 (Fig. 5D). After day 12, TENG-treated wounds were completely recovered (≤11%), while the relative wound closure area for the control and w/o a-TENG groups was still ⁓31%. Hence, it was inferred that the pulsed DC output from the a-TENG directed the cells unidirectionally toward the center of the wound from the edges, resulting in enhanced wound closure along the EF direction. Furthermore, we investigated the role of thermocatalyst Bi_2_Te_3_ NPs in the self-powered wound repair process, in which ES and a temperature gradient were simultaneously applied to activate the wound dressing. As shown in fig. S16 (A and B), no obvious difference in wound contraction was observed between the a-TENG (10.8%) and a-TENG+ Δ*T* (12.1%) groups, implying that the thermocatalyst did not contribute to wound healing. Thus, it was deduced that ES from the a-TENG is mainly responsible for the repair of normal skin wounds.

To validate the efficiency of TENG-based ES for wound tissue regeneration, histological assessment was carried out by collecting tissue samples from the wound site on day 12. The hematoxylin and eosin (H&E) staining images shown in Fig. 5E indicate the existence of a scab (green arrow) on the wound area for the untreated control and w/o TENG groups, whereas scab formation is not evident for the a-TENG group. Moreover, the TENG group showed an intact epidermis (black arrow) around the regenerated tissues, which was ~2.8 times thicker than that in the control and w/o a-TENG groups (Fig. 5F). Furthermore, Masson’s trichrome staining was performed to analyze the collagen deposition (blue-stained area) in the dermal layer of the wounds, which is an important indicator of tissue remodeling. High density of orderly arranged collagen fibers (yellow arrow) in the dermal layer was observed in wounds treated with ES (Fig. 5E). Quantitative analysis revealed that the a-TENG–based ES enhanced the collagen deposition by ~2.6 times compared to other control groups (Fig. 5G). The electrical output from TENG up-regulates the production of collagen, a key structural constituent of granulation tissue, which strengthens the ECM of the newly formed connective tissue. In addition, compared to the control groups, a-TENG treatment increased the fluorescence intensity of CD31 by ~2.7-fold, which further supports the occurrence of ES-induced enhanced angiogenesis and rapid wound recovery (Fig. 5E and fig. S17). These analytical data demonstrate that ES from the a-TENG promotes cellular responses such as fibroblast proliferation and migration and amplifies angiogenesis whichgreatly acceleratesthe biological process of wound repair relative to that of the non-stimulated groups.

### Hybrid treatment strategy for infected wound healing

**Fig. 6.**
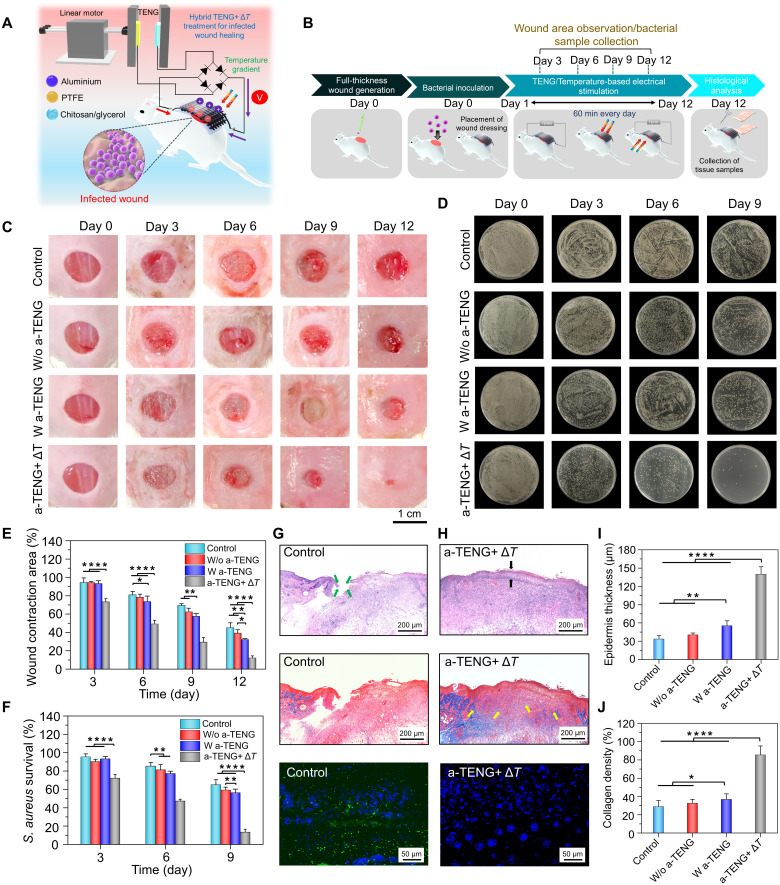
Hybrid self-powered treatment for the healing of *S. aureus*–infected wounds. (**A**) Schematic representation of wound dressing stimulated by a-TENG and thermocatalyst for healing infected wounds. (**B**) Schematic diagram of the experimental process throughout the 12-day period. (**C** and **D**) Digital photographs (C) and corresponding wound contraction areas (D). (**E** and **F**) Agar plating images (E) and survival rates of bacterial cells (F) collected from the wound area for different treatment groups of infected wounds taken on days 0, 3, 6, 9, and 12 for the control, w/o a-TENG, w/ a-TENG, and a-TENG+ Δ*T* groups. (**G** and **H**) H&E, Masson’s trichrome, and IL-6 staining of control (G) and a-TENG+ Δ*T* (H) groups. (**I** and **J**) Quantitative results concerning the thickness of the epidermis (I) and the collagen density (J) in wounds on day 12. Results are plotted as means ± SD (*n* = 6); *P < 0.05, **P < 0.01, ***P< 0.001, and ****P < 0.0001.

In addition to the electroactive layer, the hybrid wound dressing was functionalized with thermocatalytic Bi_2_Te_3_ NPs that displayed remarkable antibacterial efficiency in vitro. Hence, the wearable self-powered dressing was further used as an in situ platform to treat bacteria-infected wounds (Fig. 6A). A full-thickness wound (diameter, 8 mm) was created on the backs of the mice and incubated with *S. aureus* [1 × 10^6^ colony-forming units (CFUs)/ml] to induce infection. After 24 hours, the wound infection model was established, and the wound dressing was placed at the infectious site with the two electrodes touching the edges of the wound bed (Fig. 6B). To trigger the desired function of the hybrid dressing, different stimuli were provided, and their effect on the microbial inhibition and wound repair process was examined. The TENG groups showed a ~32.1% relative wound closure area, which was three times lower than that in normal wounds after day 12 (Fig. 6C). Moreover, the healing performance of the electrically stimulated wound dressing was almost the same as that in the control and w/o a-TENG groups. The poor healing performance of the a-TENG group toward infected wounds emphasized the need for activating the thermocatalyst in the wound dressing. In contrast to those in the control (45.1%), w/o a-TENG (39.1%), and a-TENG (32.2%) groups, wounds treated under dual stimuli (a-TENG+ Δ*T*) recovered fully on the 12th day (Fig. 6D). Moreover, the antimicrobial performance of the wound dressings was assessed by culturing the biofluids swabbed from the wound site before and after treatment through standard lysogeny broth (LB) agar plating methods (Fig. 6E). Representative colony formation assays revealed that the hybrid treatment strategy (i.e., a-TENG+ Δ*T*) resulted in the inhibition of ⁓86.7% of *S. aureus* on day 9; however, the control (~64.9%), w/o a-TENG (~59.2%), and a-TENG (~56.3%) groups displayed substantial bacterial growth (Fig. 6F). Complete eradication of *S. aureus* was achieved on day 12 in the a-TENG+ Δ*T* group. Although the thermal gradient primarily contributed to *S. aureus* elimination from the wound site, a negligible effect was observed in wound healing (figs. S18 and S19). Similarly, the wound dressing under hybrid a-TENG+ Δ*T* treatment was also most effective against *E. coli*–infected wounds (figs. S20 and S21). Therefore, it is imperative to trigger thermocatalytic and triboelectric layers at the same time to initiate microbial inhibition via H_2_O_2_ generation and promote tissue regeneration by ES from the TENG.

Furthermore, histological evaluation was performed on tissue sections excised from *S. aureus*–infected wounds before and after treatment with different strategies. The H&E staining images and its quantitative analysis reveal that the thickness of the epidermis formed in the wound treated with a-TENG+ Δ*T* was notably higher (139.8 μm) than that in the control (33.4 μm), w/o a-TENG (40.3 μm), and a-TENG (55.3 μm) groups (Fig. 6, G to I). Rapid healing of infected wounds under simultaneous temperature gradient and ES was also attributed to the higher density of collagen fiber deposition in the dermal layer, as quantified by Masson’s trichrome staining (Fig. 6, G and H, and fig. S22). Under the hybrid (a-TENG+ Δ*T*) treatment, the collagen deposition was increased by ~1.8 times compared to only w/ a-TENG group (Fig. 6I). Similarly, thicker epidermis and higher density of collagen deposition in the dermal layer was also observed for the *E. coli*–infected wounds after undergoing the hybrid treatment compared to other groups (fig. S23). In addition, the severity of wound infection is often associated with the increased secretion of pro-inflammatory factors, such as interleukin-6 (IL-6) and tumor necrosis factor–α (TNF-α). Particularly, IL-6 is considered as an important marker for diagnosis of clinical bacterial infections (*61*). Hence, immunofluorescence staining of IL-6 and TNF-α was performed to evaluate the infection levels and healing status of the wounds. As shown in Fig. 6 (G and H) and fig. S24, the expression of IL-6 was reduced by ~1.7 times under hybrid treatment, compared to the control groups, which suggests reduced infection promoting the wound recovery. Similarly, negligible expression of TNF-α was also observed for the wounds treated with a-TENG+ Δ*T* strategy (fig. S25). The comparative analysis indicated that the percentage of reduction of TNF-α and IL-6 expression in our study by day 9 is similar to the recently reported literatures (*62*, *63*). For instance, Guo *et al.* in 2021 have reported that their microneedle-based wound dressing can reduce the IL-6 secretion by 1.8 times by day 9. Moreover, increased wound infection levels were associated with the increased secretion of IL-6 and TNF-α, whereas reduced infection levels led to the negligible expression of such pro-inflammatory factors, which is also evident from our results. Furthermore, fluorescence intensity of CD31 under hybrid treatment was 2.45, which was notably higher than the control groups (fig. S26), suggesting that higher neovascularization facilitated enhanced angiogenesis. The superior antimicrobial action and wound healing rate are ascribed to thermocatalytic Bi_2_Te_3_ NPs and TENG-driven ES, respectively. All these results confirmed that the integrated wound dressing repaired the infected wound by inhibiting bacterial growth via thermocatalysts, followed by on-demand treatment by ES from the a-TENG, which promoted angiogenesis and tissue regeneration, resulting in superior healing performance. The controllable nature of the self-powered wound dressing was demonstrated, and the treatment strategy can be reconfigured on the basis of the wound status.

## DISCUSSION

In summary, we developed a fully integrated stimuli-responsive wearable wound dressing with a highly controllable treatment pathway that is capable of healing normal and infected wounds in a self-powered manner. The dressing platform was fabricated as a double-layered stacked architecture using two CFFs, which served as the substrate and electrode layers to maintain conformal contact with the wound. The entire structure was encapsulated within a chitosan hydrogel because of its extreme biocompatibility and moisture retention ability. The multiplexed functionalities of the as-prepared dressing were demonstrated by modifying Bi_2_Te_3_ NPs onto the CFF, which can be self-activated by the patient’s body-mediated temperature difference leading to in situ H_2_O_2_ generation and corresponding antibacterial activity. On the other hand, the dressing can also be triggered to generate a local EF around the wound by connecting the integrated electrodes to an a-TENG for accelerated wound healing. Under an applied temperature difference of 15°C in vitro, functionalized Bi_2_Te_3_ NPs generated ~4.4 μM H_2_O_2_, which effectively decreased the survival rates of *E. coli* and *S. aureus* to 12.7 and 18.7%, respectively, in 30 min. The generation of H_2_O_2_ by the integrated wound dressing is easily controlled by manipulating the treatment time, resulting in controlled antibacterial activity, which is beneficial when the extent of infection in the wound bed is unknown. Moreover, the ES effect of the a-TENG was analyzed by in vitro and in vivo tests. The in vitro results confirmed that a-TENG–driven ES promotes fibroblast activation by enhancing cell proliferation and migration by ~2.1- and ~3.2-fold, respectively, compared to the non-stimulated groups. Animal studies were conducted by applying the wound dressing to versatile wounds to demonstrate its configurable healing pathway. For normal skin wounds, ES-mediated therapy was followed by connecting the dressing electrodes to the a-TENG, whereas in the case of infected wounds, a hybrid treatment strategy with the TENG and temperature gradient was implemented by simultaneously triggering the Bi_2_Te_3_ NPs and chitosan/CFF, which helped combat the bacteria and subsequently heal the infected wounds. The pulsed ES from the a-TENG accelerated wound closure by about threefold for both normal and infected wounds compared to those in the control groups. The obtained results confirm the controllable characteristic of the smart dressing, where each of the functional layers can be turned on/off by activating the external stimulus from the patient’s body, thereby opening the possibility of using the dressing for a wide variety of wound types. Moreover, the as-fabricated wound dressing is simple, highly stable against environmental changes, adaptable, cost effective, customizable, and can be used for a longer course of treatment without frequent replacement, in contrast to existing dressings. In addition, the dressing can be easily scaled for its utilization in larger wounds, such as in swine or human models, suggesting the possibility of commercialization. We believe that the designed wound dressing represents the next generation of personalized wound care devices that can provide user-friendly and noninvasive therapy solutions to address the unmet need of patients with chronic wounds. Furthermore, this self-activated wound dressing represents a promising therapeutic platform for other pathological disorders such as venous ulcers, ischemic wounds, and keloid scarring.

## MATERIALS AND METHODS

### Preparation of chitosan hydrogel

To prepare the chitosan hydrogel, chitosan powder (low molecular weight, 1 g) was dissolved in aqueous acetic acid (40 ml). The solution was stirred at room temperature for 12 hours to obtain a homogeneous viscous mixture. Then, glycerol (0.8 ml) and polyethylene glycol (molecular weight, 4000; 0.8 g) were added to the solution and continuously stirred at 50°C for 10 min. Next, the mixture (18 ml) was cross-linked using 1-ethyl-3-(3-dimethylaminopropyl) carbodiimide (95 mg, 25 mM) and *N*-hydroxysuccinimide (30 mg, 12.5 mM) at room temperature. The amidation cross-linking reaction between amino and carboxyl groups of neighboring chitosan molecules leads to the formation of a gel-like structure, which was used in this study.

### Synthesis of Bi_2_Te_3_ NPs

First, a stock solution was prepared by dissolving sodium hydroxide (0.8 g) in 10 ml of ethylene glycol at 45°C for 2 hours. Then, 10 ml of the stock solution was added to bismuth nitrate pentahydrate (0.1 g), sodium telluride (0.067 g), and polyvinylpyrrolidone (0.235 g) loaded into a 25-ml three-neck flask. The mixture was continuously stirred at room temperature for 10 min. Afterward, the three-neck flask was placed in a water bath at 45°C for 20 min. Next, the three-neck flask was transferred to an oil bath at 190°C and stirred for 3 hours until complete dissolution. After the reaction, 10 ml of acetone and 30 ml of ethanol were added to the solution, and the mixture was centrifuged at 6700*g* for 10 min. The supernatant was discarded, and the process was repeated three times. Last, the filtered Bi_2_Te_3_ NPs were redispersed in 30 ml of ethanol and were used for further experiments.

### Preparation of the wound dressing

The wound dressing consists of a thermocatalytic layer, upper and bottom electrode layers, and an encapsulation layer. First, the surface of the CFF electrodes was ultrasonically cleaned in acetone, isopropanol, and deionized water for 5 min each. Next, the fabric electrodes were dried in a hot air oven at 60°C for 20 min. Then, 20 μl of Bi_2_Te_3_ NPs was drop-cast onto the bottom CFF electrode layer (1 cm by 1 cm) and dried at 60°C for 30 s. A spacer layer (PET) was placed on top of the bottom electrode layer to prevent immediate contact with the top electrode. Then, another CFF of the same dimensions was integrated together as the upper electrode layer with the Bi_2_Te_3_ NP–coated electrode layer. Next, 1.2 ml of as-prepared chitosan hydrogel solution was coated on the outer side of both the top and bottom electrode layers to encapsulate the dressing. The uncoated part of the top and bottom fabric electrodes placed outside the wound area was used as the two electrode terminals, which were connected to the two ends of the TENG for ES experiments.

### Characterization

The morphology of the Bi_2_Te_3_ NPs was analyzed by using high-resolution TEM (JEOL, JEM-F200). XRD (Rigaku, TTRAX III) was used to investigate the structural properties of the prepared nanomaterials, such as phase purity and crystallinity. Amplitude-modulated KPFM (Bruker icon ScanAsyst) equipped with a thermal stage was used to monitor the surface potential distribution of the Bi_2_Te_3_ NPs. Furthermore, the morphological characterization of the chitosan hydrogel was performed by using FESEM (JEOL, JSM-7600F). The elemental distributions of the Bi_2_Te_3_ NPs and chitosan hydrogel film were investigated by using EDX (Oxford INCA). The adhesiveness of wound dressing at different RHs was assessed using a lap shear testing method. First, the wound dressings (1 cm by 1 cm) were placed into the humidity chamber at different RHs from 20 to 80% for 30 min. For the adhesive strength measurement, the treated wound dressing was placed between the two pieces of precleaned porcine skin (4 cm by 2 cm) with a bonding area of 1 cm by 1 cm and was clamped to the tensile machine. The adhesive strength was measured by a Universal Tensile Tester (Jun Yen Machinery Co. Ltd., CY-6040A1) in tensile mode at a rate of 5 mm/min. The point at which the two pieces of skin detaches from each other is denoted as the adhesive strength.

### Detection of H_2_O_2_ generated by Bi_2_Te_3_ NPs

The generation of H_2_O_2_ was measured by using the Amplex Red reagent assay. Generally, Amplex Red reacts with H_2_O_2_ in the presence of horseradish peroxidase (HRP) to produce a red-fluorescent oxidation product, i.e., resorufin. Before detection, a stock solution of Amplex Red was prepared by dissolving 0.4 mg of the powder in 3.1 ml of dimethyl sulfoxide. In addition, 0.5 mg of HRP was dissolved in phosphate-buffered saline (PBS; pH 5.8) to prepare the stock solution. The as-prepared wound dressing containing Bi_2_Te_3_ NPs was placed in 1 ml of sodium chloride (0.85% NaCl) solution and subjected to different temperature gradients (−15°, 0°, 7°, and 15°C) by using a hot plate. At each temperature gradient, the solution was collected at different time intervals (5, 10, 15, and 30 min) to evaluate the time-dependent H_2_O_2_ generation. After time-dependent temperature treatment, the solution was filtered through a 0.2-μm polyvinylidene fluoride membrane filter to remove residual impurities. Then, 270 μl of filtrate solution was added to a mixture of 30 μl of Amplex Red solution and 3 μl of HRP solution. The resulting solution was incubated at room temperature for 30 min under dark conditions. The fluorescence intensity (excitation, 530 nm) of the samples was measured in the emission range of 560 to 750 nm using a photoluminescence spectrophotometer (HITACHI, F-7000).

For determining the humidity-dependent H_2_O_2_ generation, the wound dressing (1 cm by 1 cm) was immersed in 1 ml of 0.85% NaCl solution and subjected to different temperature gradients (−15°, 0°, 7°, and 15°C) by using a hot plate. The entire setup was placed inside a humidity chamber, and the different humidity conditions were achieved by purging nitrogen gas inside the chamber. At each humidity condition, the solution was collected at different time intervals (5, 10, 15, and 30 min) to evaluate the time-dependent H_2_O_2_ generation for each temperature gradient. The same process for the H_2_O_2_ detection as described above was followed for measuring the production of H_2_O_2_ at different RHs.

### Preparation of the bacterial culture

*E. coli* K12 and *S. aureus* cells were grown in LB medium at 37°C for 16 hours in an incubator. After 16 hours, the *E. coli* K12 and *S. aureus* cells were diluted to obtain an optical density of 0.06 and 0.3, respectively, at 670 nm. The bacterial cell solutions were centrifuged twice at 4000 rpm for 10 min, and the supernatant was discarded. The bacterial cells in the pellet were resuspended in 0.85% NaCl to obtain a final concentration of 2 × 10^8^ CFU/ml for the antibacterial studies.

### In vitro antibacterial activity of wound dressing

For the antibacterial experiments, the wound dressing was subjected to a temperature gradient to activate the thermocatalytic Bi_2_Te_3_ NPs encapsulated within the wound dressing. The wound dressing (1 cm by 1 cm) was immersed in 1 ml of bacterial solution (2 × 10^6^ CFU/ml) on a hot plate. To establish the temperature gradient, the bottom side of the wound dressing was allowed to react at specific temperatures (7°C/29°C/37°C) adjusted on the hot plate, and the upper side of the wound dressing reacted with room temperature (22°C). Control experiments were performed by immersing the dressing in bacterial solution at room temperature without generating any temperature gradient. Aliquots of 100 μl of bacterial solution were collected from each group at different time intervals (5, 10, 15, and 30 min) and plated on aseptic agar plates to check for antibacterial activity. The agar plates were incubated at 37°C for 24 hours. After 24 hours, the survival rates were calculated by the following formula:

$$\text{Bacteria\textbackslash{} survival}\;(\%)=\frac{C}{C_{0}}\times 100\%$$

where *C*_0_ is the concentration of the bacterial solution before temperature treatment and *C* is the remaining concentration of bacteria after temperature treatment.

The viability of the bacterial cells was further determined using a live/dead bacterial staining kit (LIVE/DEAD BacLight Bacterial Viability Kit, L7012) containing PI and SYTO 9 dye. After temperature treatment, 100 μl of bacterial solution from each group was collected at different time intervals (5, 10, 15, and 30 min) and added to 100 μl of solution containing SYTO 9 and PI. The mixture was kept at room temperature for 15 min in the dark under vigorous shaking. The fluorescence intensities of SYTO 9 (excitation, 475 nm; emission, 530 nm) and PI (excitation, 475 nm; emission, 640 nm) were recorded separately and merged using ImageJ software.

### Fabrication of an a-TENG

An a-TENG was fabricated using PTFE and chitosan/glycerol film as triboelectric layers. To prepare the chitosan/glycerol layer, glycerol was added to a solution containing 2 weight% chitosan powder dissolved in acetic acid. The constituents were stirred at room temperature until a homogeneous mixture was obtained. Then, the chitosan/glycerol layer was coated on a silicon substrate with nanostructured patterns by spin coating. Last, the as-prepared sample was incubated at 60°C for 4 hours in a hot air oven to form a chitosan/glycerol thin film with nanostructures. The chitosan/glycerol film was coated on top of a highly transparent and flexible PET sheet (2 cm by 2 cm), which served as the triboelectric and electrode layers. A conductive aluminum film (thickness, 100 nm) was sputtered onto another PET sheet of the same dimensions. PTFE was used to cover the top of the PET/Al sheet, which formed the other triboelectric layer. Then, two copper wires were connected to the aluminum film and chitosan/glycerol film as the electric output leads of the TENG. The arch-shaped structure was formed by gluing the sides of the two triboelectric layers using Kapton tape.

### Characterization and electrical measurement of TENG

The structural morphology of the chitosan/glycerol film was observed using FESEM. For the electrical measurements, an external force was applied to the TENG via a commercial linear motor. The output voltage and current were measured by a programmable electrometer (Keithley, 6514).

### In vitro cell culture

NIH-3T3 fibroblast cells were obtained from the Food Industry Research and Development Institute in Taiwan. Cells were cultured in Dulbecco’s modified Eagle’s medium supplemented with 10% fetal bovine serum and 1% penicillin-streptomycin solution at 37°C in 5% CO_2_ in a humidified incubator.

### Cytotoxicity study of the wound dressing

The cytotoxicity evaluation was conducted by directly immersing the wound dressing in the cell medium. First, cells were seeded in 48-well plates at a density of 2.5 × 10^4^ cells per well. After culturing for 12 hours, the wound dressing was cut into 1 cm–by–1 cm pieces and immersed into the cell medium. Control groups were cultured with medium only. Then, the cells were cultured for 24 hours at 37°C in 5% CO_2_ in a humidified incubator. The viability of the cells after being in direct contact with the wound dressing was examined by the alamarBlue (alamarBlue Cell Viability Reagent, DAL1025) assay. After being cocultured, the cell medium and the wound dressing were removed and replaced with 200 μl of fresh medium. Then, 20 μl of alamarBlue reagent was added to each well, and the plates were incubated for 4 hours at 37°C in a 5% CO_2_ incubator. Last, the absorbance was measured at a wavelength of 570 nm using a microplate reader (Molecular Devices, SpectraMax, iD5) according to the manufacturer’s instructions.

### Cell proliferation and migration assays

For the cell proliferation assay, cells were seeded in 96-well cell culture plates at a density of 3 × 10^3^ cells per well and cultured for 12 hours. Two vertically aligned gold (Au foil) electrodes (30 mm by 160 mm) were placed in each well to create the ES effect. The electrodes were connected to the two terminals of the TENG, generating an output voltage of 25 V at 1 Hz. Control experiments were also performed by only placing the electrodes in the cell wells without connecting to the TENG. After 72 hours of ES, the cell proliferation rate was evaluated with a Cell Counting Kit-8 assay by measuring the optical absorbance at a wavelength of 492 nm using a microplate reader according to the manufacturer’s protocol. Furthermore, cells from different groups before and after ES were stained with Calcein-AM dye and imaged using an inverted fluorescence microscope (Olympus, IX83).

For the cell migration assay, cells were seeded in six-well cell plates at a density of 1 × 10^6^ cells per well and cultured for 12 hours. After the cells reached confluence, a straight scratch was created in the middle of each well using a sterilized 100-μl sterilized pipette tip. The cells were then washed twice with the cell medium to remove the detached cells. Two vertically aligned Au foil electrodes (30 mm by 160 mm) were placed in each well such that the direction of the EF was parallel to the direction of cell migration. The electrodes were connected to the two terminals of the a-TENG controlled by a linear motor, generating an output voltage of 25 V at 1 Hz. Control experiments were also performed by only placing the electrodes in the cell wells without making a connection to the TENG. The migration of the cells was monitored every 12 hours by taking optical images using an inverted fluorescence microscope (Olympus, IX83). The migration area was quantified from the images using ImageJ software. The rate of migration was expressed as the cell migration ratio, which is defined as the ratio of the change in scratch area to the initial scratch area at different time intervals:

$$\text{Cell\textbackslash{} migration\textbackslash{} ratio}\;(\%)=\frac{A_{0}-A_{t}}{A_{0}}\times 100\%$$

where *A*_0_ and *A*_t_ are the areas of the initial scratch area and healing scratch area at different time intervals, respectively.

### In vivo wound healing assay

All animal experiments were approved by the Institutional Animal Care and Use Committee (number 109037) and were performed according to the guidelines following Animal Protection Law by the Council of Agriculture. Thirty 6-week-old female BALB/c mice were obtained from the National Laboratory Animal Centre, Nangang, Taiwan and were randomly divided into different groups, with *n* = 6 in each group. The animals were first anesthetized by inhalation of anesthesia, which was initially induced by 2 to 5% isoflurane and maintained with 2% isoflurane. Then, the dorsal surface of the mice was shaved and sterilized with alcohol. A full-thickness round wound (diameter, 8 mm) was created on the dorsal region of the mice using a biopsy punch. Then, the as-prepared wound dressing (1 cm by 1 cm) was placed over the wound area, and a commercial 1 cm by 1 cm of Tegaderm (3M, 1624WB) film was used to firmly attach the dressing to the wound site. The uncoated part of the top and bottom electrodes was placed outside the wound area and were then connected to the two terminals of the a-TENG, which was controlled by the linear motor. During the experiment, the TENG generated an output voltage of 25 V while operating at a frequency of 1 Hz. For the temperature-only group, an ice pack was placed at the top of the dressing to generate a temperature difference. Wounds covered with commercial Tegaderm (3M, 1624WB) film and untreated dressing were the two control groups used in the experiment. The closure of the wound area was monitored at different time intervals by taking photographs on days 0, 3, 6, 9, and 12. Wound healing was quantified by calculating the wound area using ImageJ software and was expressed as follows

$$\text{Wound\textbackslash{} contraction\textbackslash{} area}\;(\%)=\frac{A_{t}}{A_{0}}\times 100\%$$

where *A*_0_ and *A*_t_ are the wound area at day 0 and the remaining wound area at different time intervals (days 3, 6, 9, and 12), respectively.

### In vivo infected wound healing assay

To establish the infected wound model, a full-thickness round wound (diameter, 8 mm) was created on the dorsal region of the mice. The wound area was then inoculated with 20 μl of *S. aureus* bacteria (1 × 10^6^ CFU/ml) solution. Afterward, the as-prepared wound dressing (1 cm by 1 cm) was placed over the wound area, and a commercial Tegaderm (3M, 1624WB) film with dimensions of 1 cm by 1 cm was used to firmly attach the dressing to the wound site. Like the normal wound healing assay, the uncoated part of the top and bottom electrodes was placed outside the wound area and were then connected to the two terminals of the a-TENG, which was controlled by the linear motor. For the thermoelectric stimulus (temperature only group), an ice pack was placed at the top of the dressing, and the bottom layer of the dressing was in direct contact with the wound area, which generated a temperature difference across the dressing. Moreover, the dual stimulus was applied by simultaneously connecting the dressing electrodes to the a-TENG and placing an ice pack on top of the dressing for a duration of 1 hour every day for a period of 12 days. Wounds covered with only commercial Tegaderm (3M, 1624WB) film and untreated dressing were the two control groups used in the experiment. Bacterial samples were collected from the wound area at different time intervals (days 0, 3, 6, 9, and 12) using a sterilized cotton swab, which was later dissolved in 1 ml of PBS buffer (pH 7.4) solution. One hundred microliters of each collected sample was added to agar plates and cultured at 37°C for 24 hours. The bacterial survival rates of *S. aureus* were calculated by using the following formula:

$$S.\;aureus\;\mathrm{survival}\;(\%)=\frac{C}{C_{0}}\times 100\%$$

where *C*_0_ is the concentration of the bacteria before treatment and *C* is the remaining concentration of the bacteria after the treatment.

Similarly, for the *E. coli*–infected wounds, the wound area was inoculated with 20 μl of *E. coli* bacteria (1 × 10^6^ CFU/ml) solution, and the wound dressing (1 cm by 1 cm) was placed over the wound area and covered with a commercial Tegaderm film with dimensions of 1 cm by 1 cm. The TENG-based ES and thermocatalytic treatment was applied in a similar manner as the *S. aureus*–infected wound for a duration of 30 min every day for 12 days. The survival rates of *E. coli* were also calculated using the same equation. Simultaneously, wound area closure was monitored at different time intervals by taking photographs on days 0, 3, 6, 9, and 12. Wound healing was quantified by calculating the wound area as a percentage of the area on day 0 using ImageJ software.

### Histological and immunohistological analysis

After 12 days of treatment, wound tissues were excised, fixed in 10% formalin buffer at room temperature for 24 hours, and embedded in paraffin to prepare histological sections. The sections were stained with H&E and Masson’s trichrome. The bright-field microscopic images of the samples were recorded using an inverted fluorescence microscope (Olympus, IX83). Similarly, the wound tissues were collected on day 12 and were immunofluorescently stained with anti-CD31 antibody (Thermo Fisher Scientific, MA3105). Furthermore, the immunofluorescence staining of TNF-α and IL-6 were carried out by excising the wound tissues on day 9 and staining with primary antibodies anti–TNF-α antibody (Abcam, ab1793) and anti–IL-6 polyclonal antibody (Bioss, bs-0782R), respectively. All the steps of immunofluorescence staining were carried out according to the manufacturer’s protocol. The fluorescence intensities were measured using inverted fluorescence microscopy (Olympus, IX83).

### Statistical analysis

All the obtained experimental results were statistically probed and are expressed as means ± SD. The difference between two groups was analyzed using the unpaired Student’s *t* test. In all cases, a *P* value <0.05 was considered to indicate a significant difference.
