# Supplementary material for: A self-powered multifunctional dressing for active infection prevention and accelerated wound healing
Source: Sci Adv. 2023 Jan 25;9(4):eadc8758. doi: 10.1126/sciadv.adc8758 (PMC9876552; doi:10.1126/sciadv.adc8758)
Supplement: Supplementary file 1 — Figs. S1 to S27 [file sciadv.adc8758_sm.pdf]

Science Advances
AAAS

Supplementary Materials for

A self-powered multifunctional dressing for active infection prevention and accelerated wound healing

Snigdha Roy Barman *et al.*

Corresponding author: Zong-Hong Lin, linzh@mx.nthu.edu.tw

Sci. Adv. **9**, eadc8758 (2023)
DOI: 10.1126/sciadv.adc8758

The PDF file includes:

Figs. S1 to S27
Legend for movie S1

Other Supplementary Material for this manuscript includes the following:

Movie S1

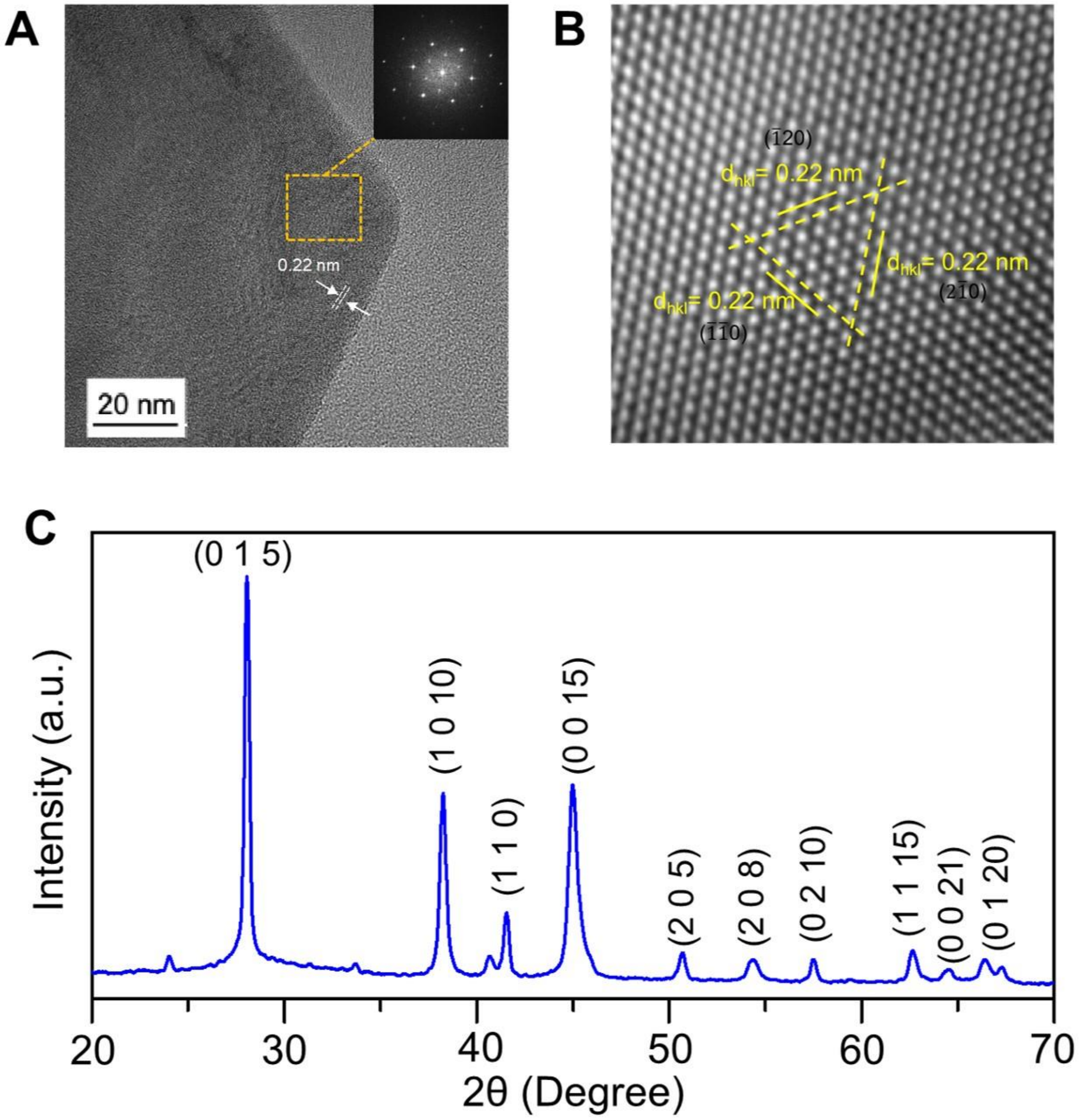

Fig. S1. Characterization of Bi_2Te_3 NPs. (A) HRTEM image and **(B)** IFFT pattern of the as-prepared Bi_2Te_3 NPs. **(C)** XRD characterization of the formed Bi_2Te_3 NPs.

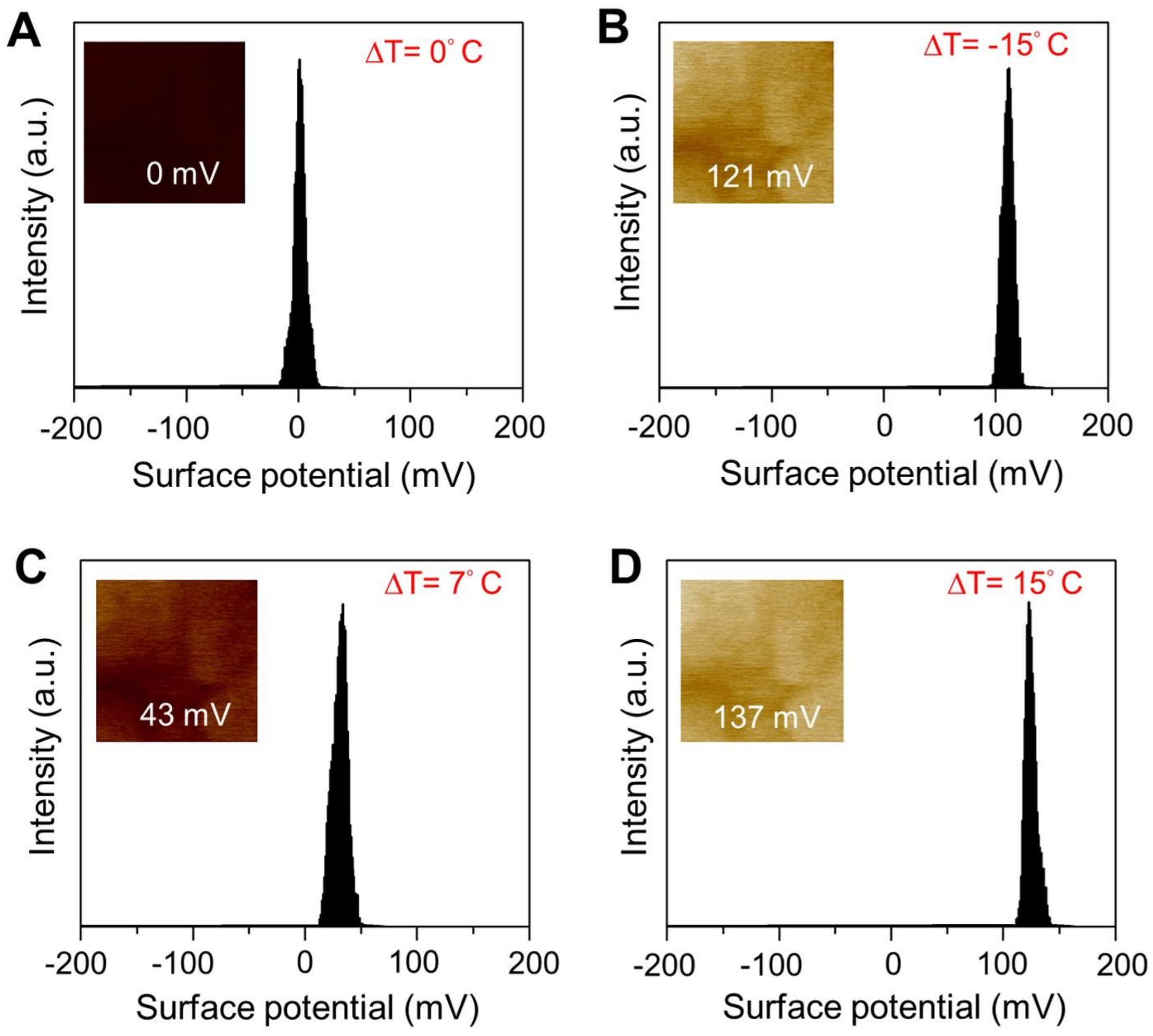

Fig. S2. Surface potential measurement of Bi_2Te_3 NPs using KPFM integrated with thermal stage at different temperature gradients. (A) 0 °C. **(B)** -15 °C. **(C)** 7 °C and **(D)** 15 °C.

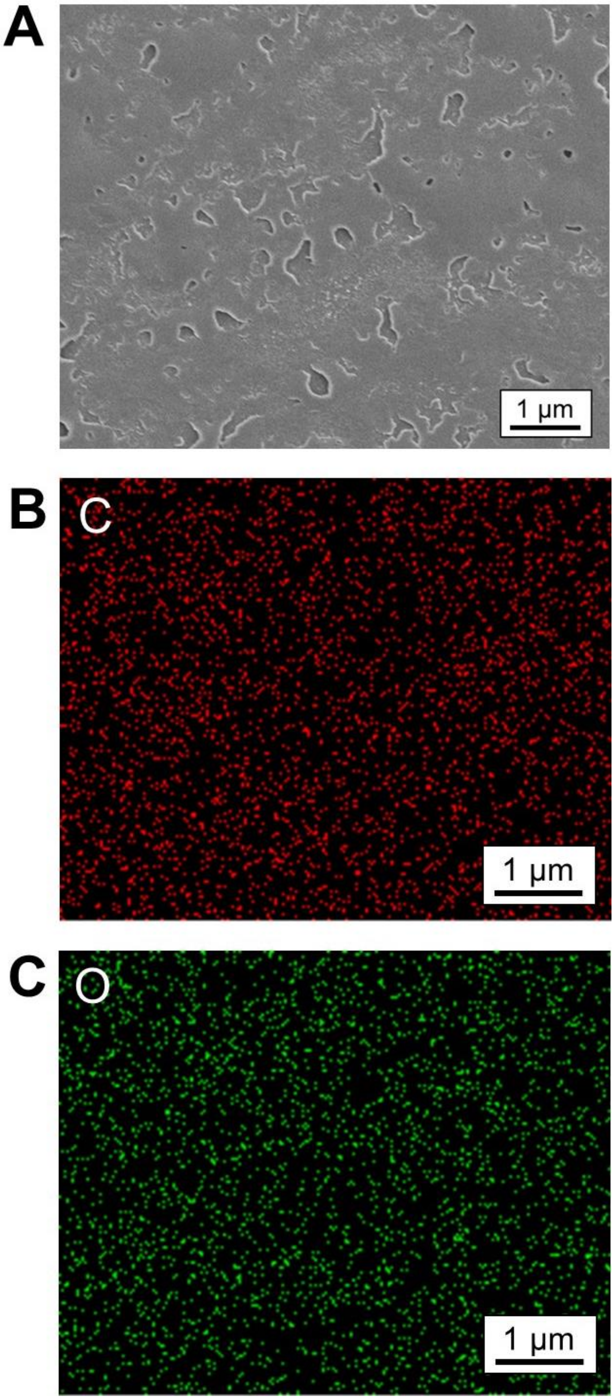

Fig. S3. Surface characterization of chitosan hydrogel. (A) FESEM image of the chitosan hydrogel. EDX mapping of chitosan hydrogel showing the distribution of **(B)** C and **(C)** O.

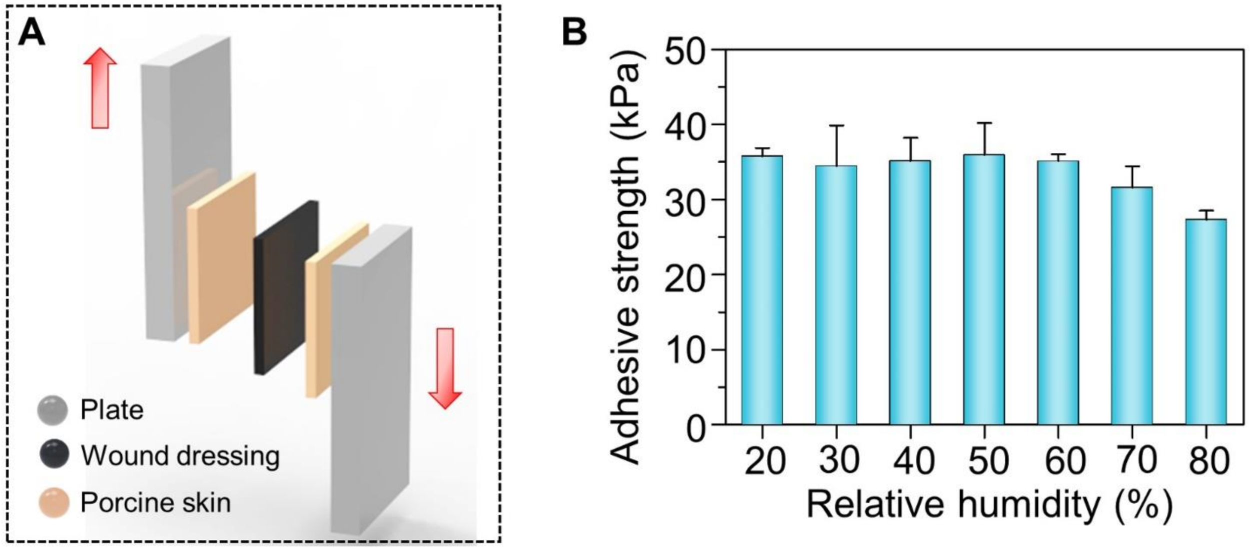

Fig. S4. Effect of relative humidity on the adhesive strength of the self-powered wound dressing. (A) Experimental set-up for the lap-shear test. **(B)** Adhesive strength obtained at different humidity conditions starting from 20% to 80%.

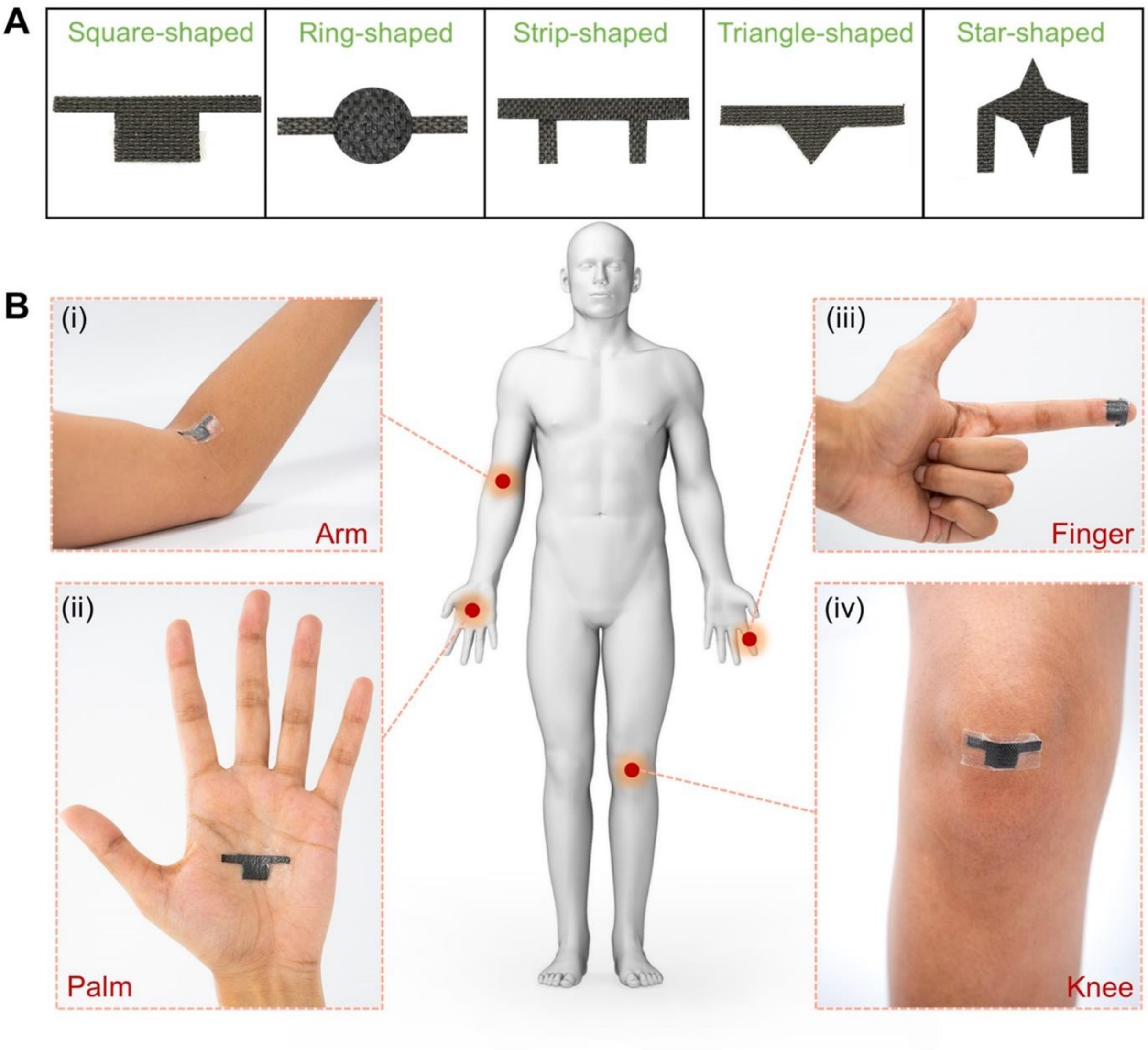

Fig. S5. Adaptable nature of the as-designed wound dressing. (A) Different morphologies of the wound dressing fabricated to fit different wound shapes. **(B)** Placement of the wound dressing at different wound sites of the human body.

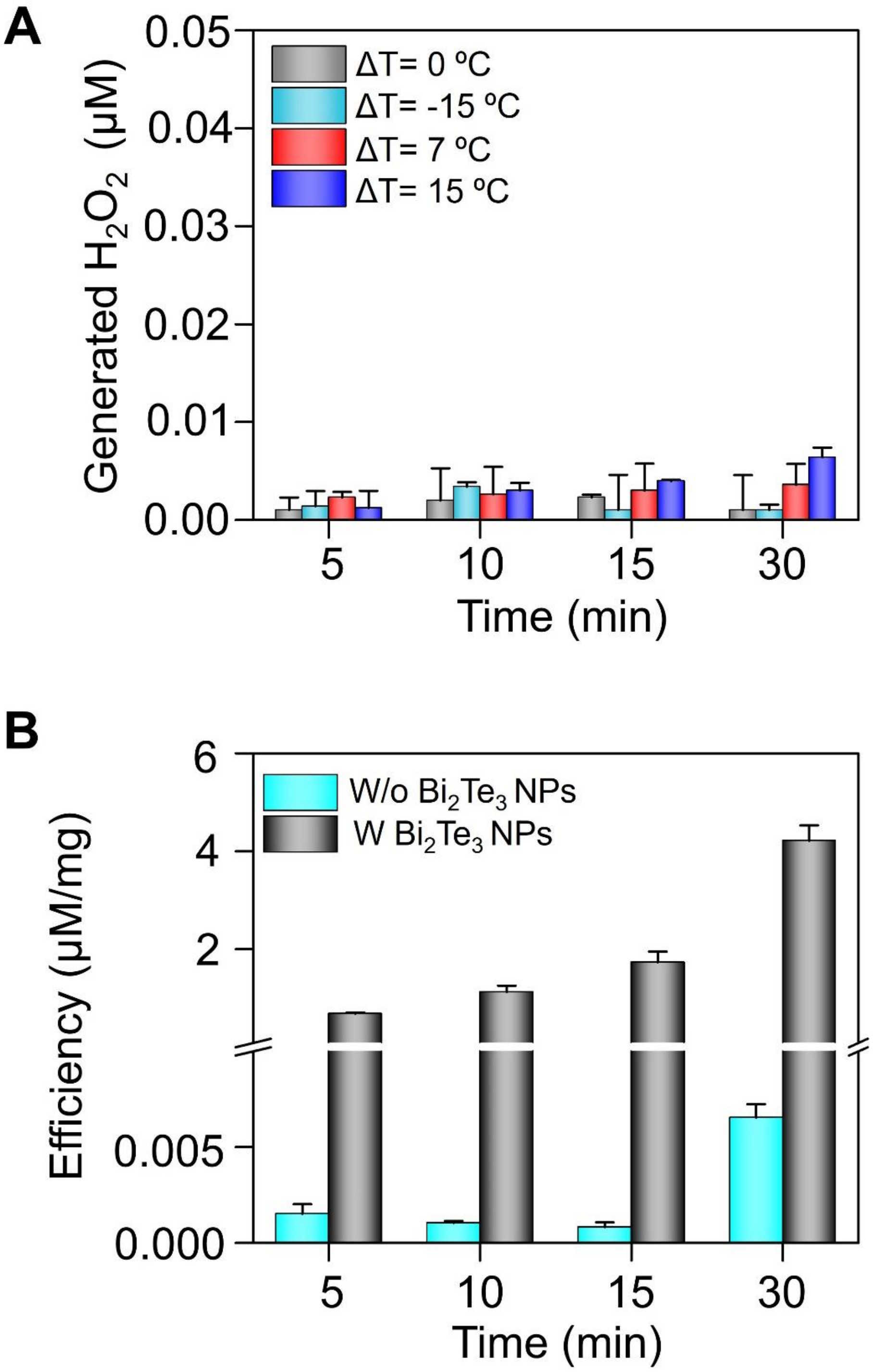

Fig. S6. Efficiency of H_2O_2 generation by the wound dressing. (A) H_2O_2 generated by control dressings not coated with Bi_2Te_3 NPs. **(B)** Comparison of the H_2O_2 generation efficiency between dressings coated with and without Bi_2Te_3 NPs.

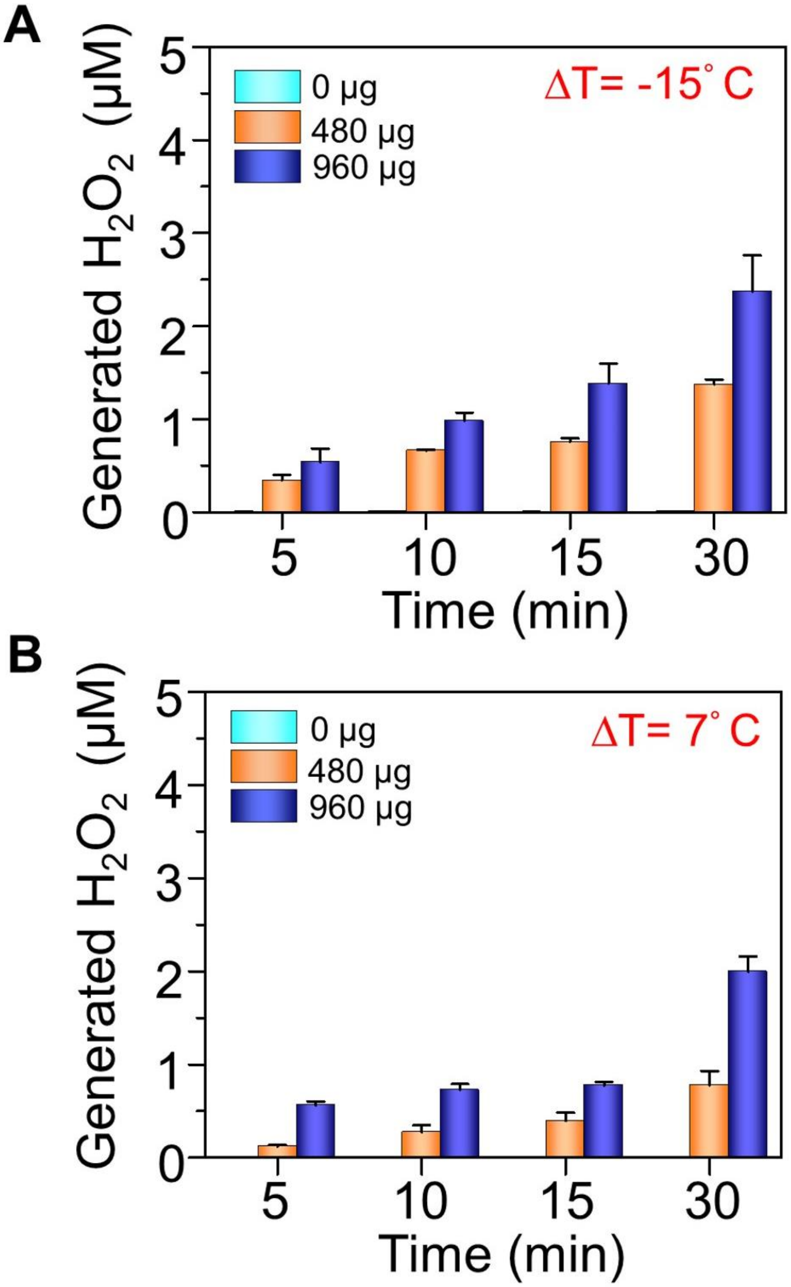

Fig. S7. Generation of H_2O_2 by dressings coated with different amounts of Bi_2Te_3 NPs at different temperature gradients. (A) -15 °C and **(B)** 7 °C.

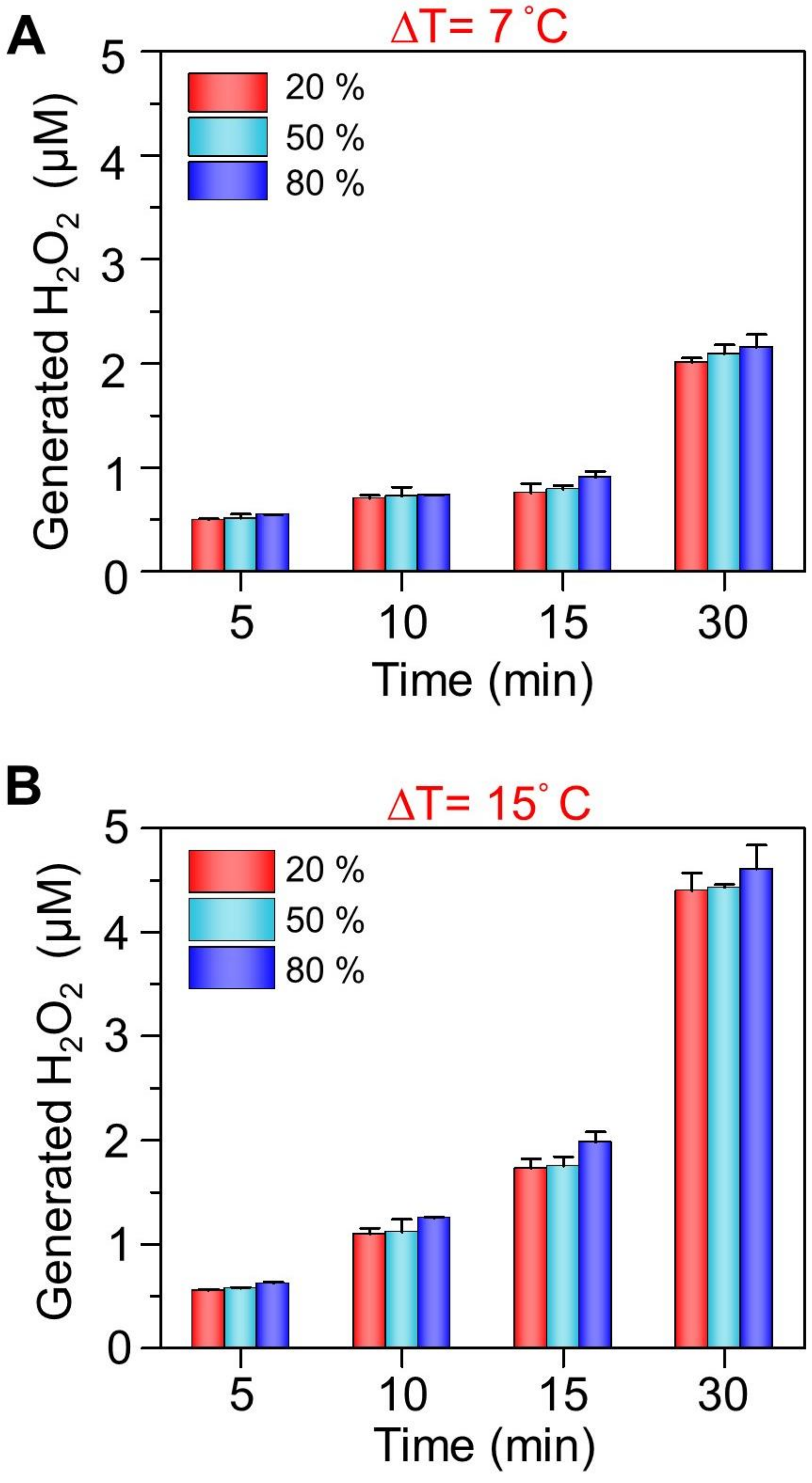

Fig. S8. Generation of H_2O_2 by the wound dressing at different humidity conditions subjected to varying temperature gradients. (A) 7 °C and **(B)** 15 °C.

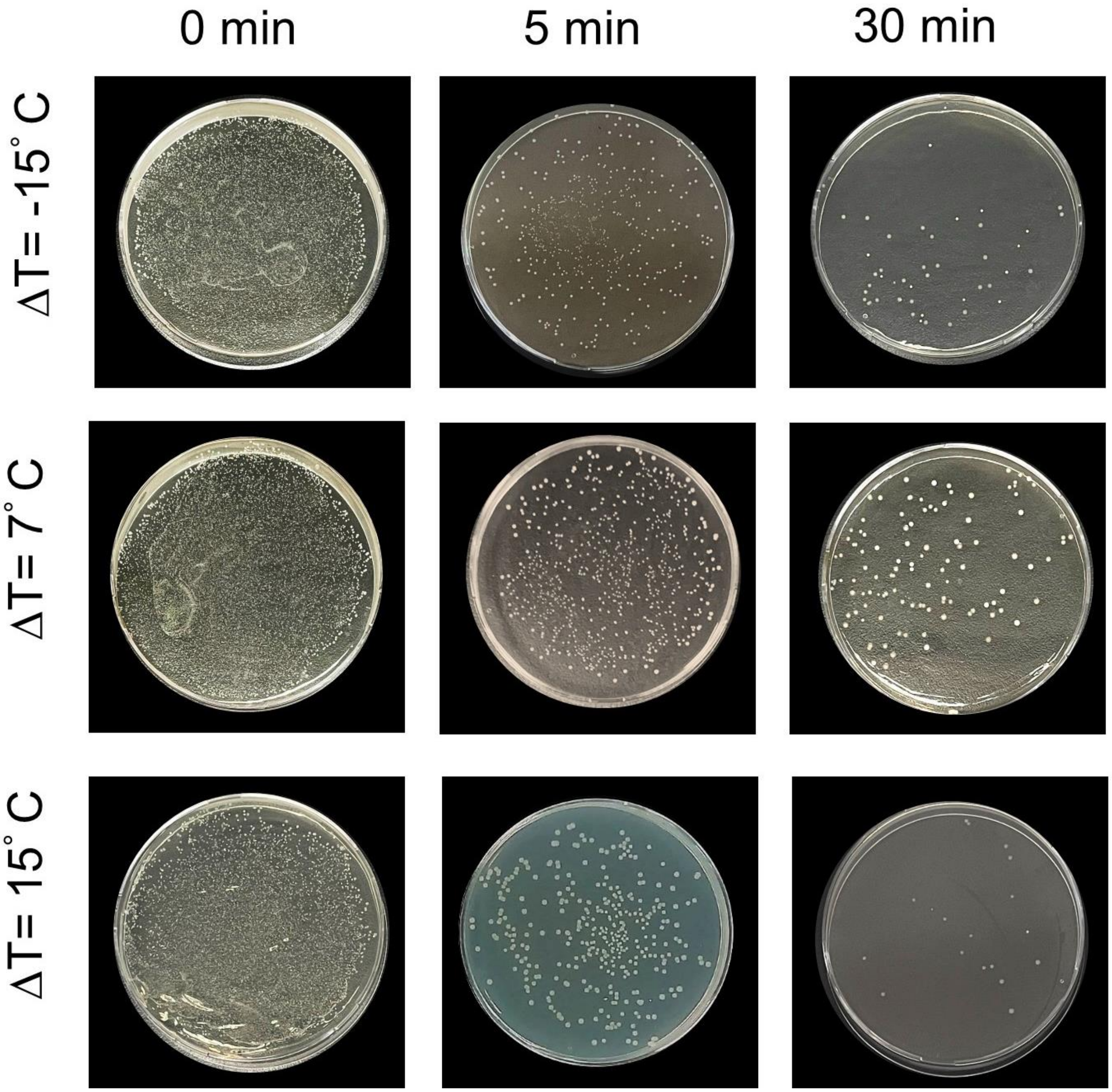

Fig. S9. Photographs of agar plates of *S. aureus* colonies at different temperature gradients collected at different time intervals.

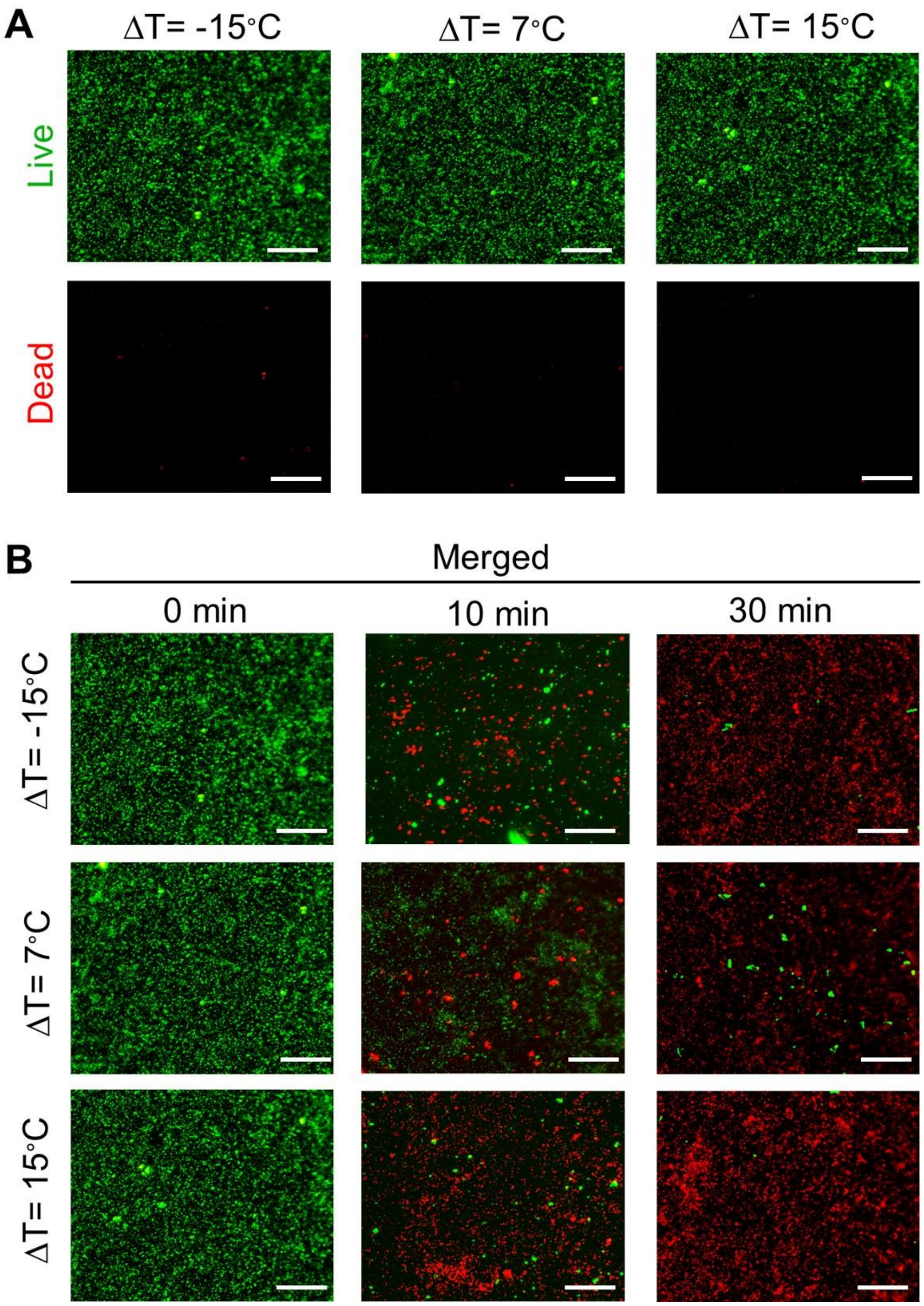

Fig. S10. Images of live (green fluorescence) and dead (red fluorescence) *E. coli* cells. (A) Staining images of live and dead bacteria at different temperature gradient collected at 0 min. **(B)** Merged images of live and dead bacteria following different temperature gradient treatments. Scale bars, 100 μm. Results are plotted as mean ± s.d. (n = 3).

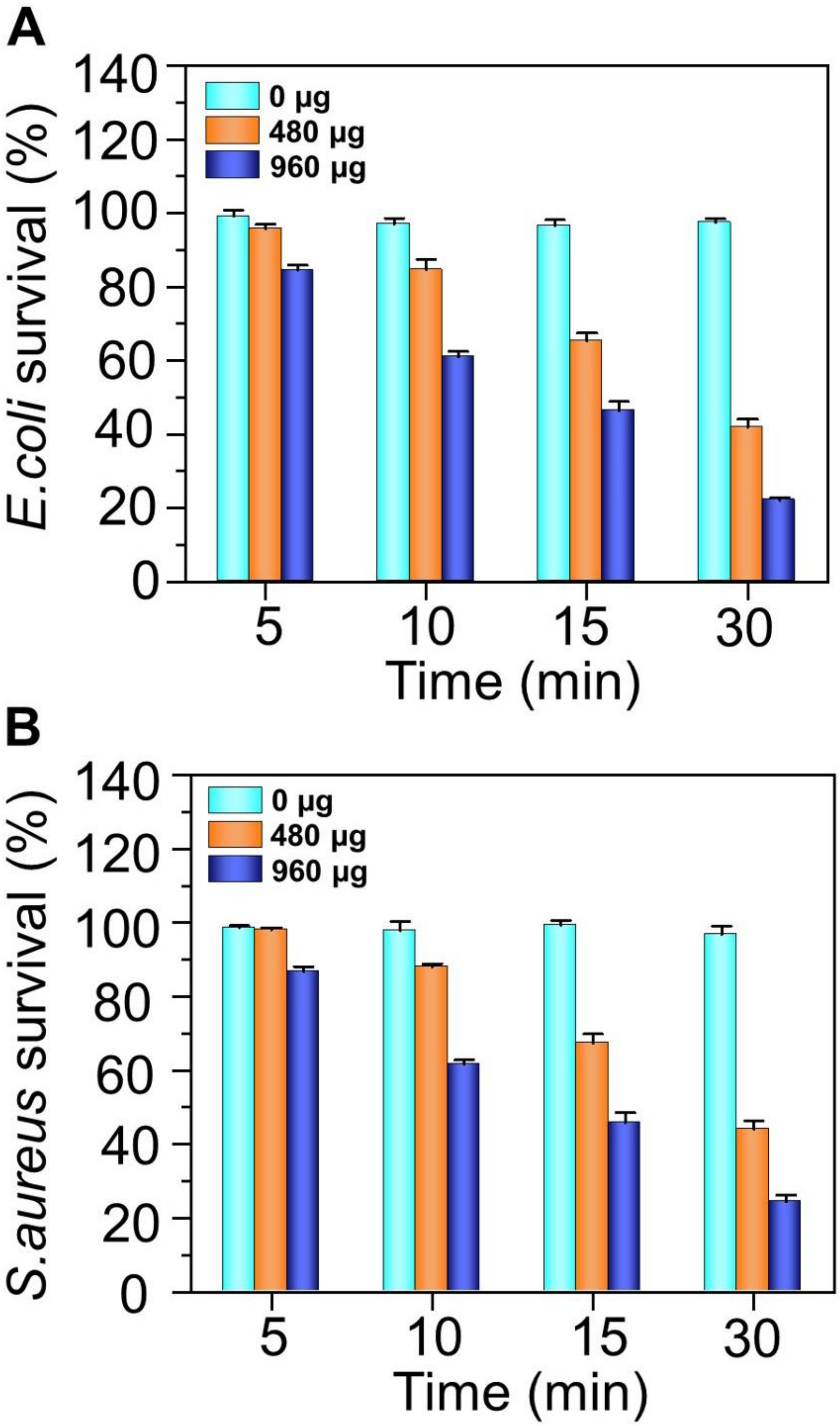

Fig. S11. Antibacterial activity of the wound dressing coated with different amounts of Bi_2Te_3 NPs at a temperature gradient of 15 °C against. (A) *E. coli* and (**B)** *S. aureus*.

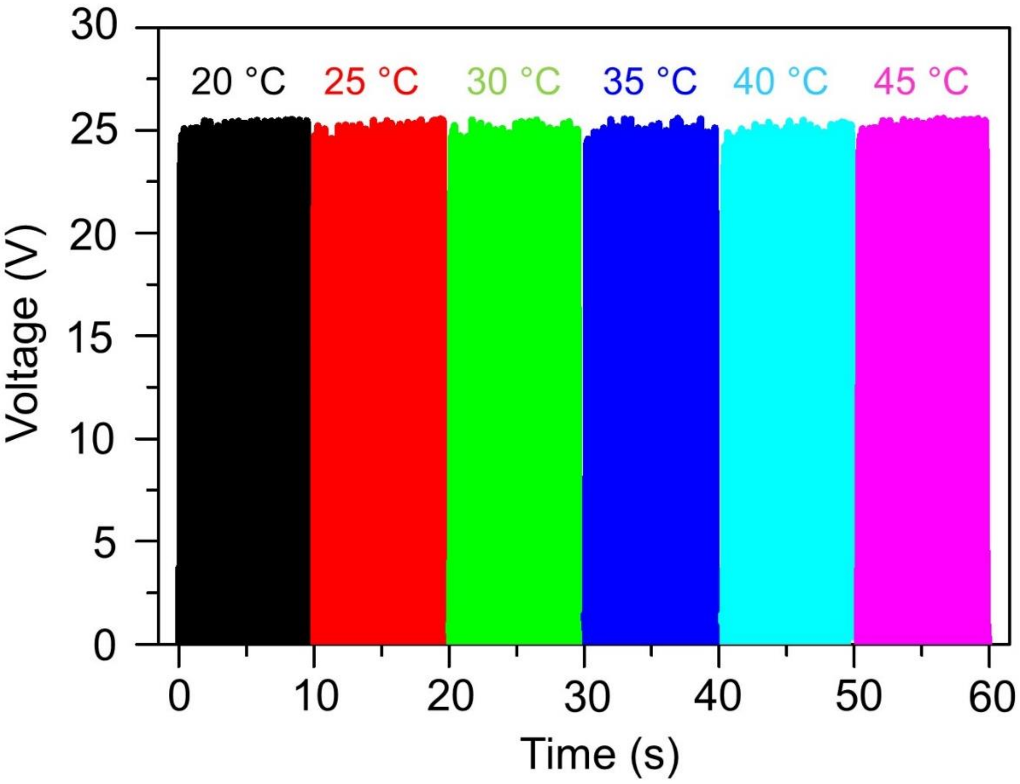

Fig. S12. Effect of environmental temperature changes on the generated output voltage of TENG.

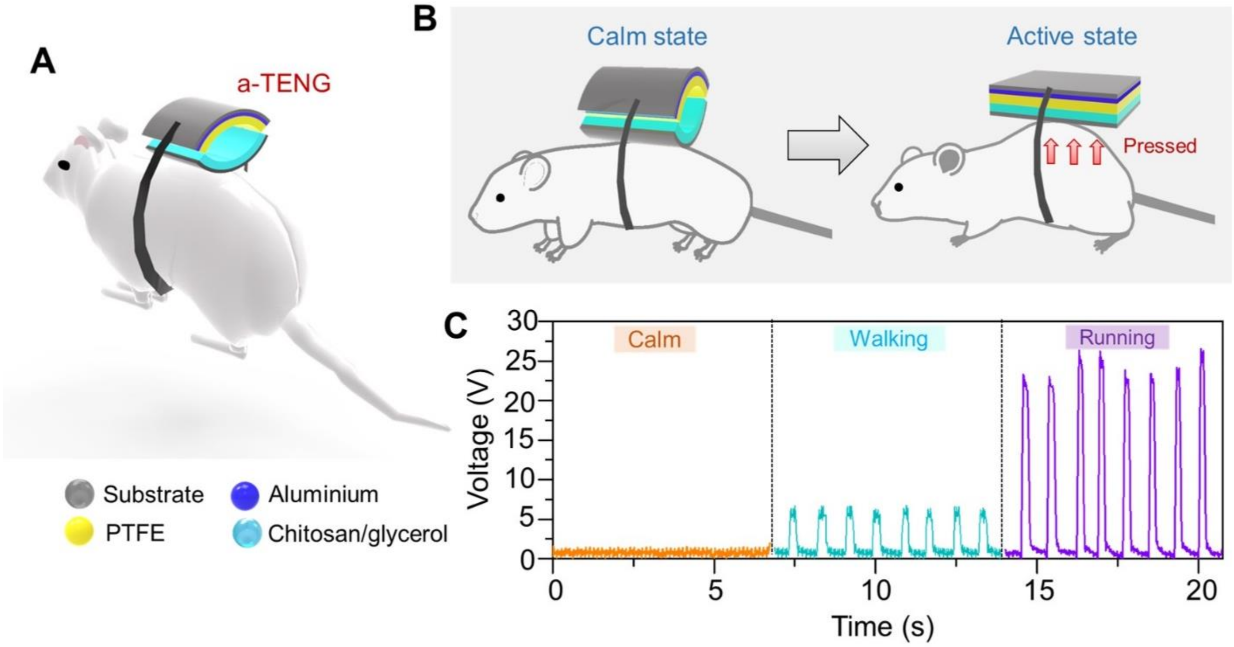

Fig. S13. Demonstration of the wearable characteristics of the wound dressing. (A) Schematic representation of a wearable a-TENG on the back of the mice. **(B)** Mechanism of contact between chitosan/glycerol film and PTFE at different stages of mouse motion. **(C)** Voltage measurement at different stages of mouse motion such as the calm, walking and running states.

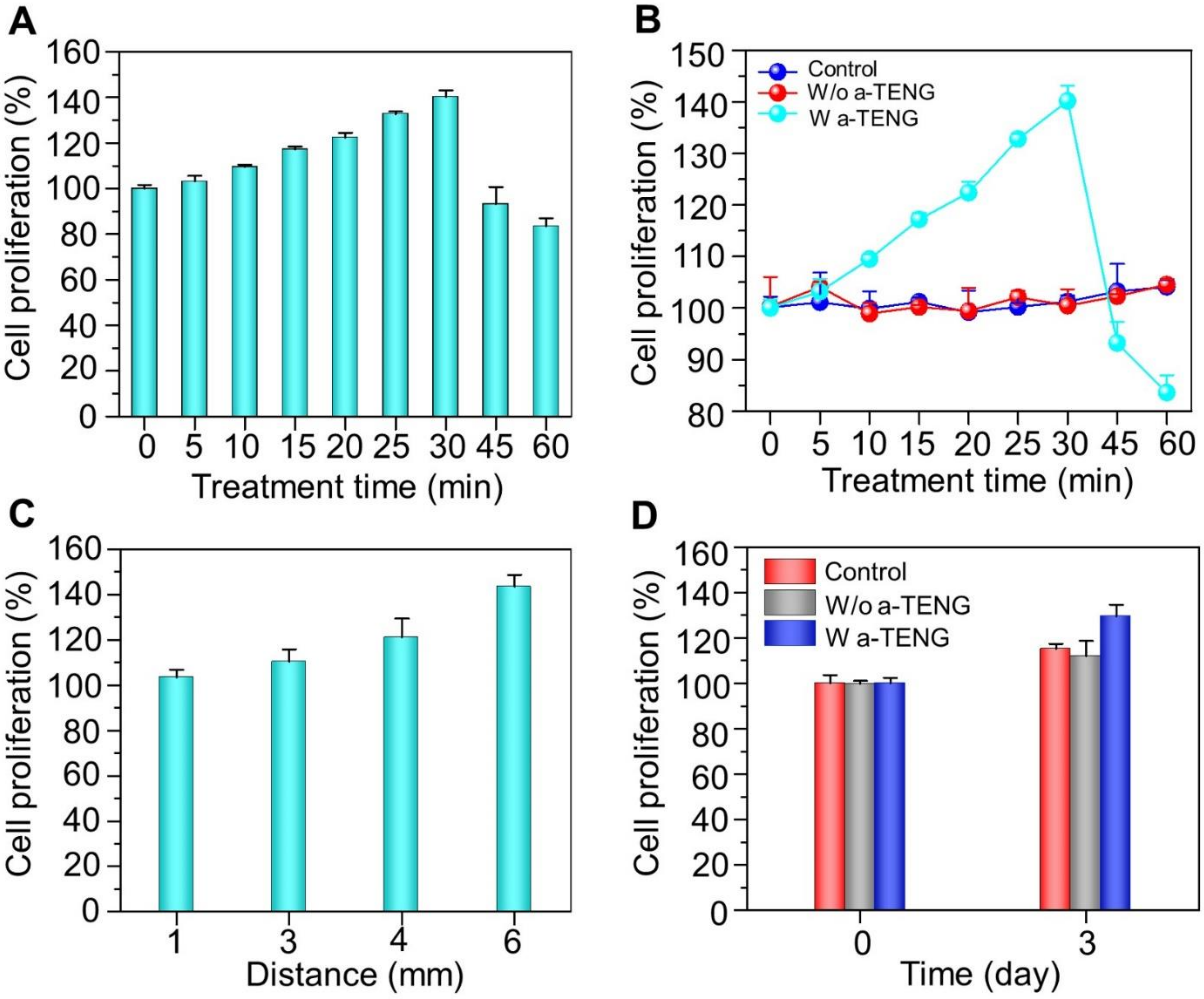

Fig. S14. Influence of different TENG based ES parameters on cellular behavior. (A) Cell proliferation under different ES times. (**B)** Comparison of cell proliferation of the a-TENG groups with that of the control groups. (**C)** Effect of the interelectrode distance on cell proliferation. **(D**) Cell proliferation of different groups when Au foil electrodes were used for ES. Results are plotted as mean ± s.d. (n = 4).

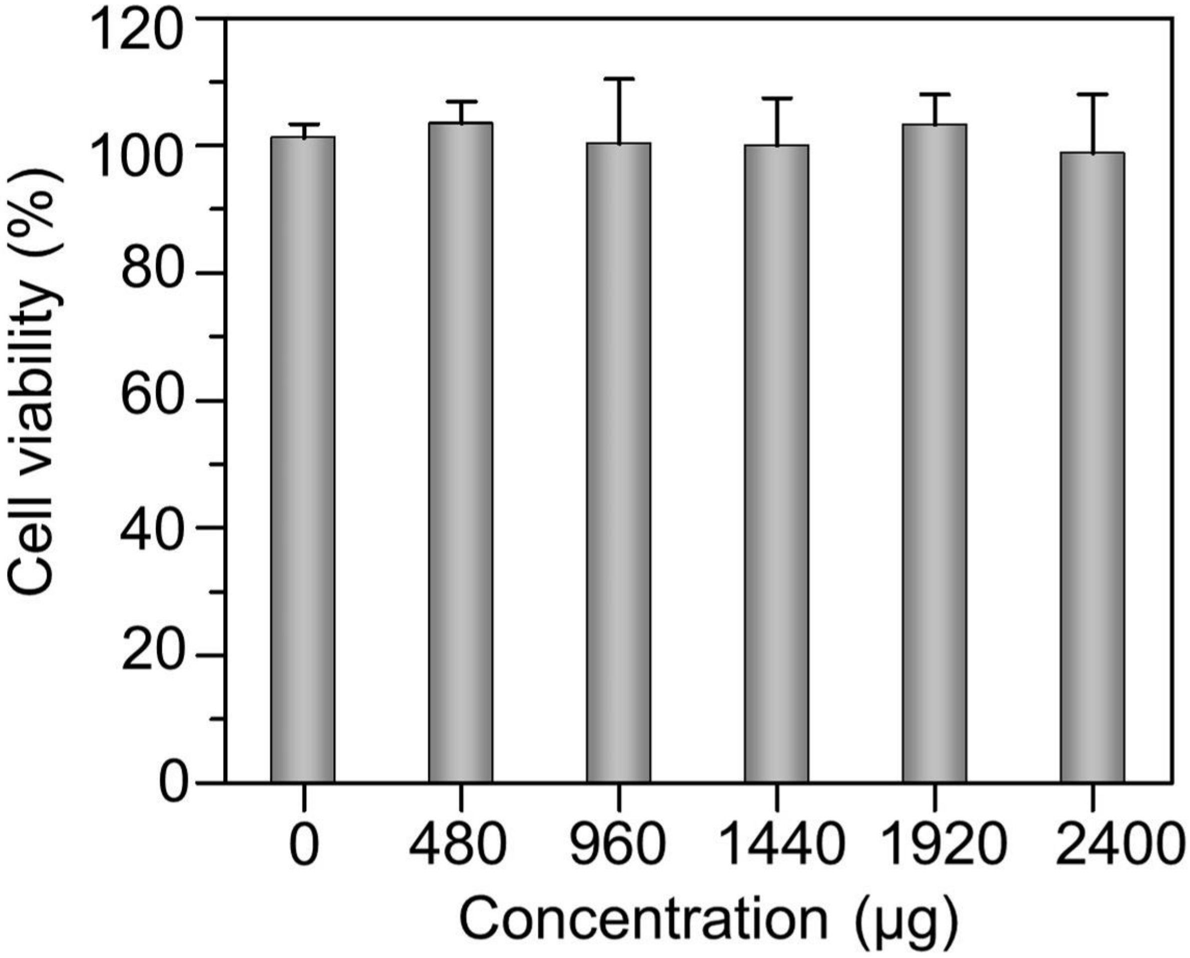

Fig. S15. Cell viability obtained after culturing the cells with different amounts of Bi_2Te_3 NPs to determine the in-vitro biocompatibility of the wound dressing.

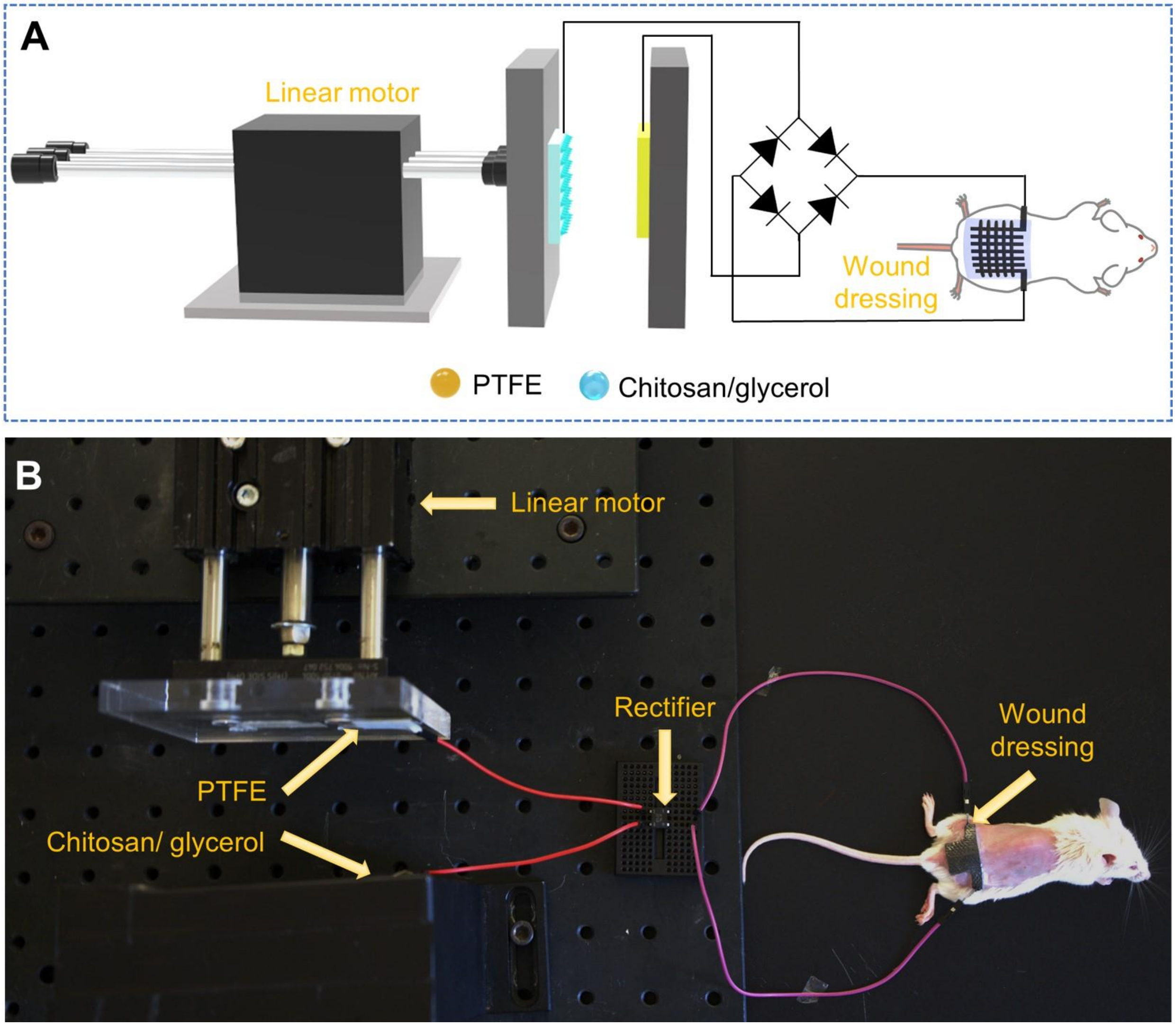

Fig. S16. Experimental setup for providing a-TENG based electrical stimulation to the wound site. (A) Schematic and **(B)** Digital photographs of connecting linear motor controlled chitosan-glycerol/PTFE based TENG to the self-powered wound dressing.

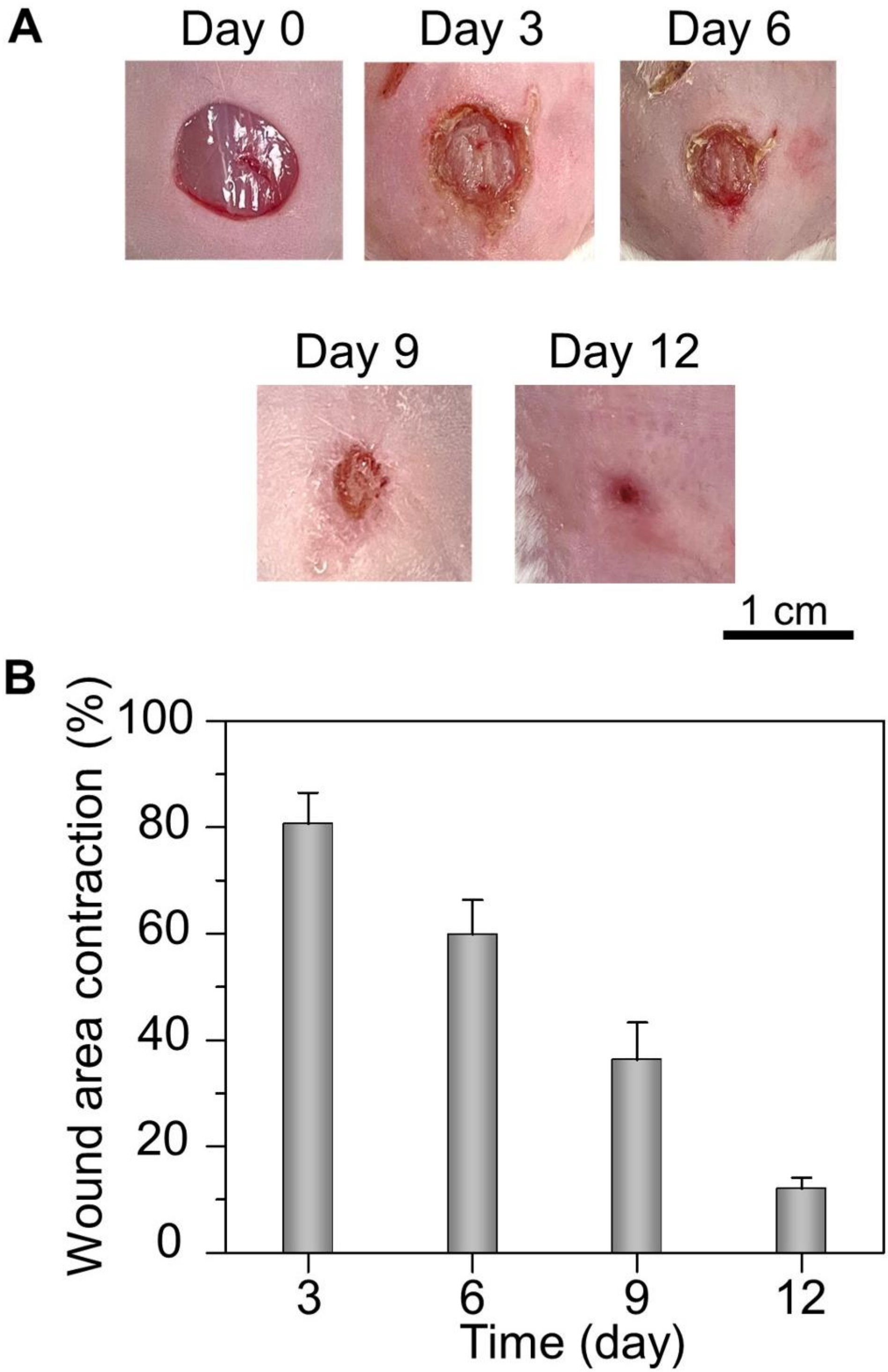

Fig. S17. Effect of hybrid a-TENG+ ΔT treatment on normal wound healing. (A) Photographs and (**B)** calculated wound contraction areas of the normal wound area after hybrid treatment at different time intervals.

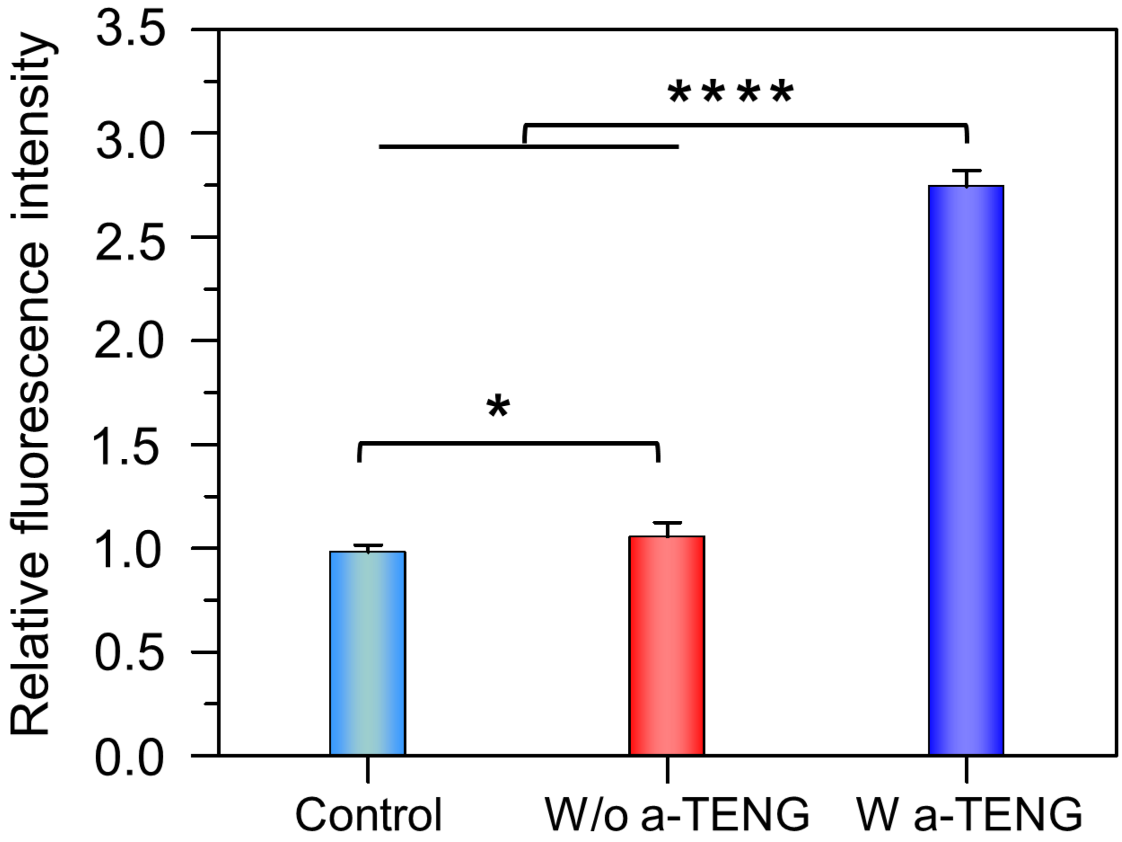

Fig. S18. Quantitative analysis of CD31 expressed in normal wounds on day 12 following different treatments. Results are plotted as mean ± s.d. (n = 6), $*p < 0.05$, $**p < 0.01$, $***p < 0.001$ and $****p < 0.0001$.

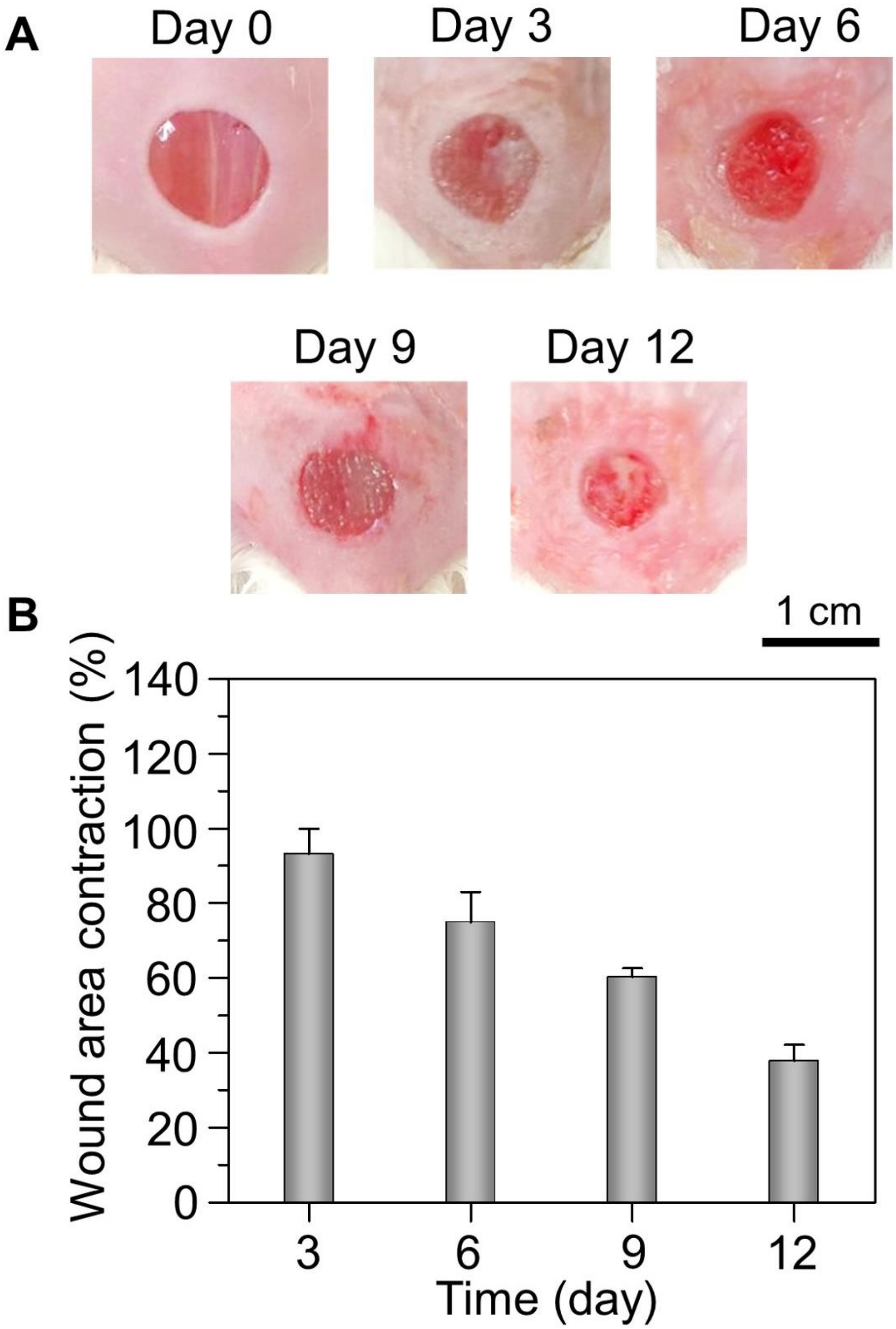

Fig. S19. Effect of thermocatalytic treatment on *S. aureus* infected wound healing. (A) Photographs and **(B)** Calculated wound contraction areas of the infected wound area treated with only a temperature gradient at different time intervals

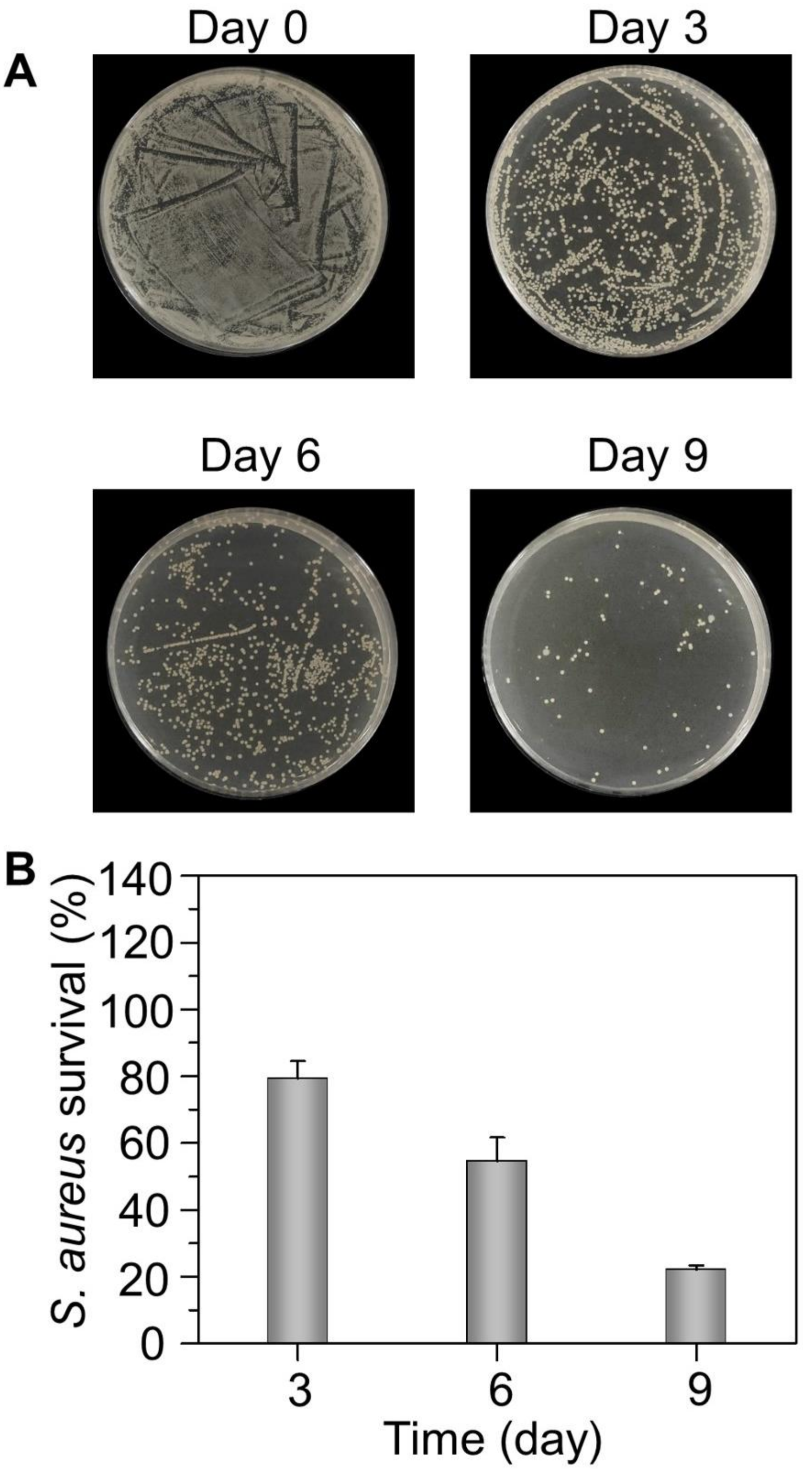

Fig. S20. In-vivo antibacterial activity of the wound dressing. (A) Photographs of agar plates and **(B)** Survival percentage of *S. aureus* colonies collected from the infected wound area after treatment with only temperature gradient.

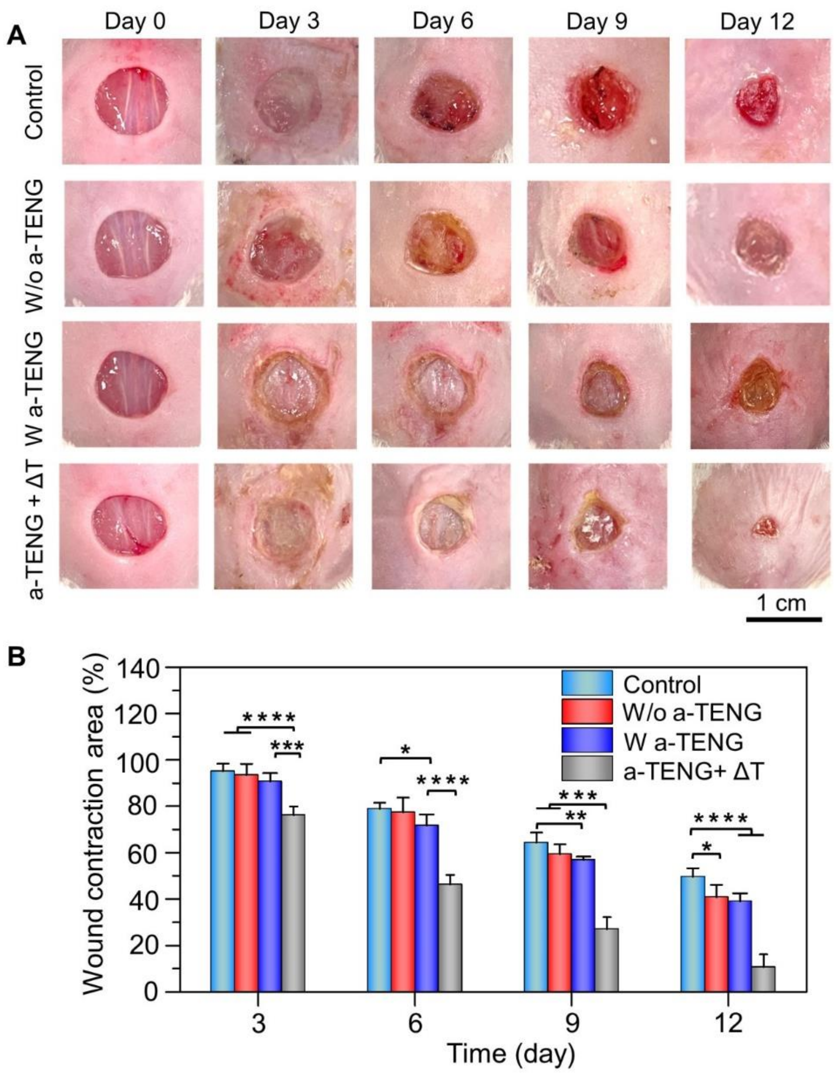

Fig. S21. In-vivo wound healing results of the *E. coli* infected wounds under different treatments. (A) Digital photographs and **(B)** Corresponding wound contraction areas on days 0, 3, 6, 9 and 12. Results are plotted as mean ± s.d. (n = 6), *$*p < 0.05$, $**p < 0.01$, $***p < 0.001$* and *$****p < 0.0001$*.

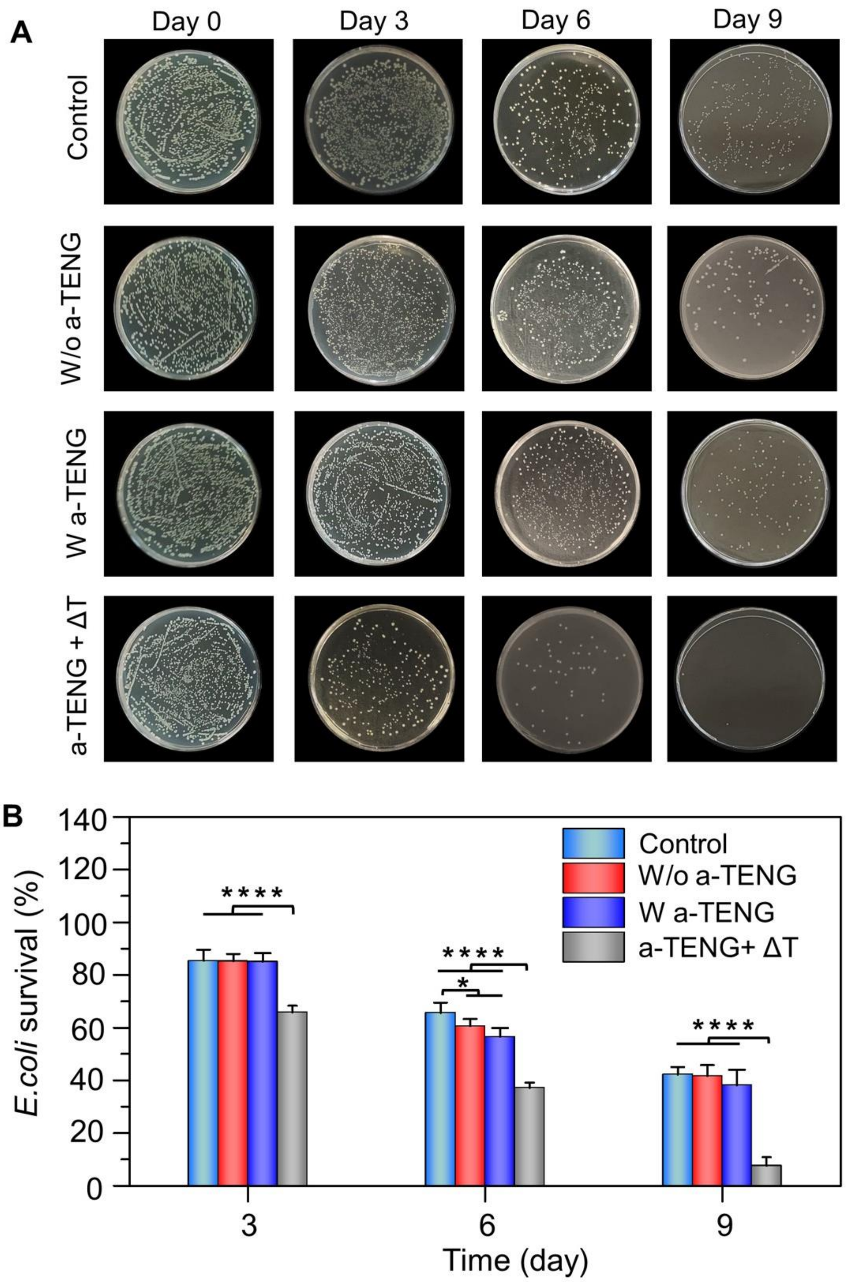

Fig. S22. In-vivo antibacterial results of the *E. coli* infected wounds under different treatments. (A) Agar plating images and **(B)** Survival rates of bacterial cells collected from the wound area for different treatment groups of infected wounds taken on days 0, 3, 6, 9 and 12. Results are plotted as mean ± s.d. (n = 6), $*p < 0.05$, $**p < 0.01$, $***p < 0.001$ and $****p < 0.0001$.

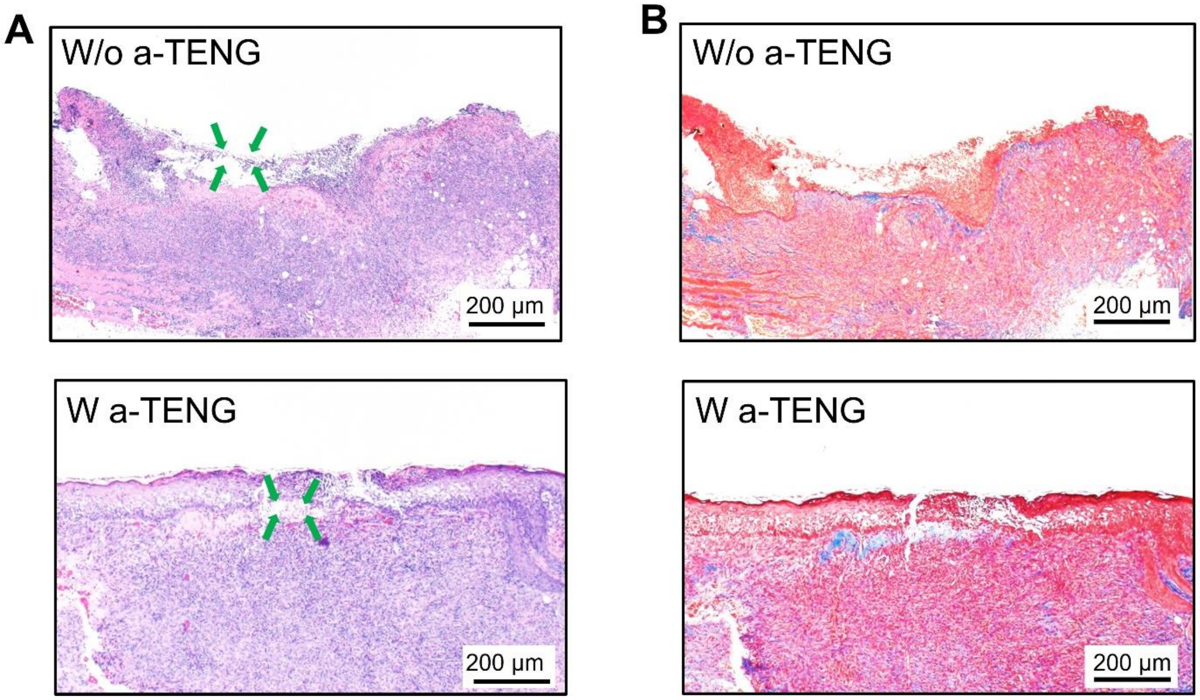

Fig. S23. Histological analysis of *S. aureus* infected wound tissues. (A) H&E and **(B)** Masson's trichrome staining images of the wound area of W/o and W a-TENG groups on day 12.

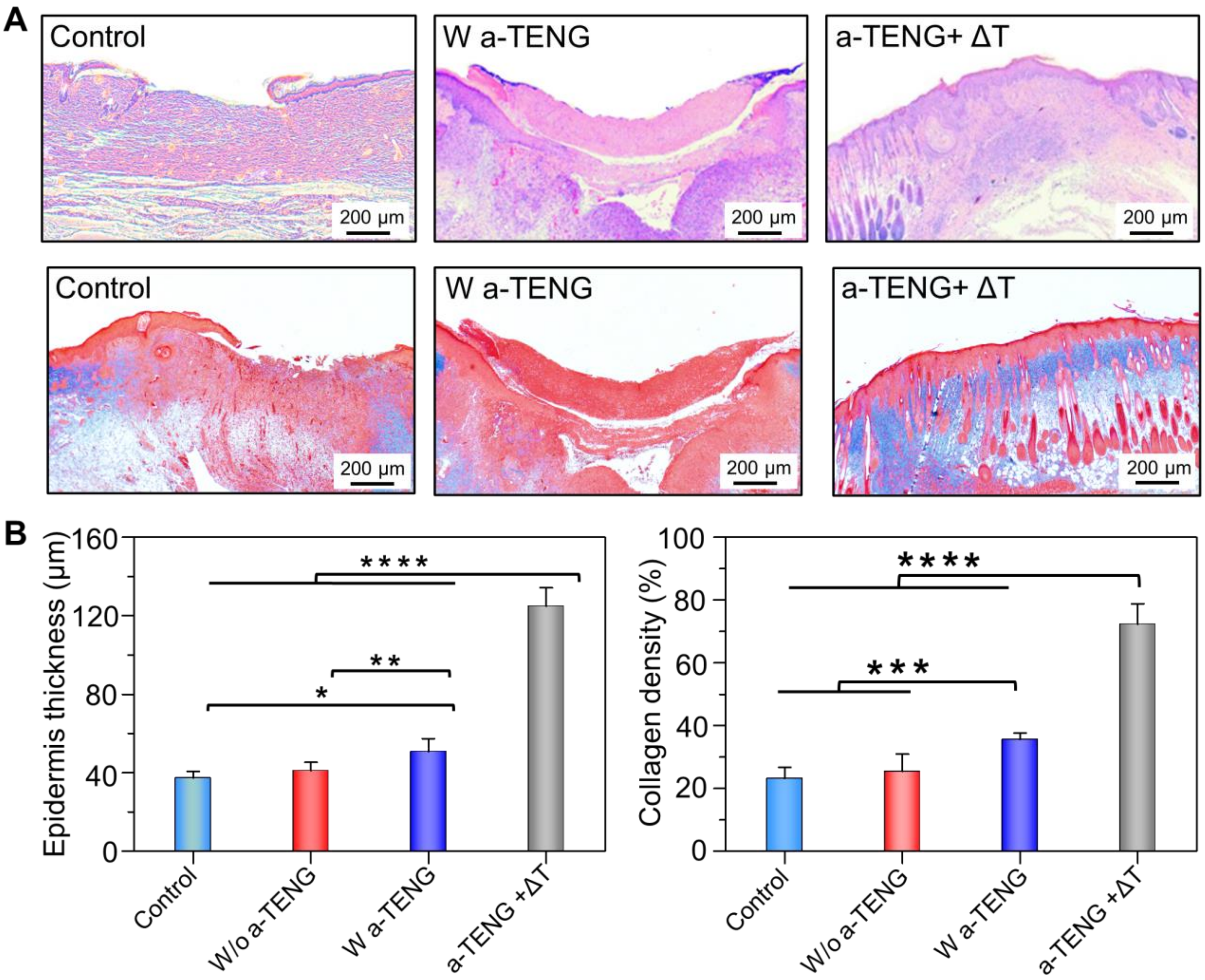

Fig. S24. Histological analysis of *E. coli* infected wound tissues. (A) H&E and Masson's trichrome staining images and **(B)** Corresponding quantitative analysis of the tissue samples collected from the wound site on day 12. Results are plotted as mean ± s.d. (n = 6), $^{*}p < 0.05$, $^{**}p < 0.01$, $^{***}p < 0.001$ and $^{****}p < 0.0001$.

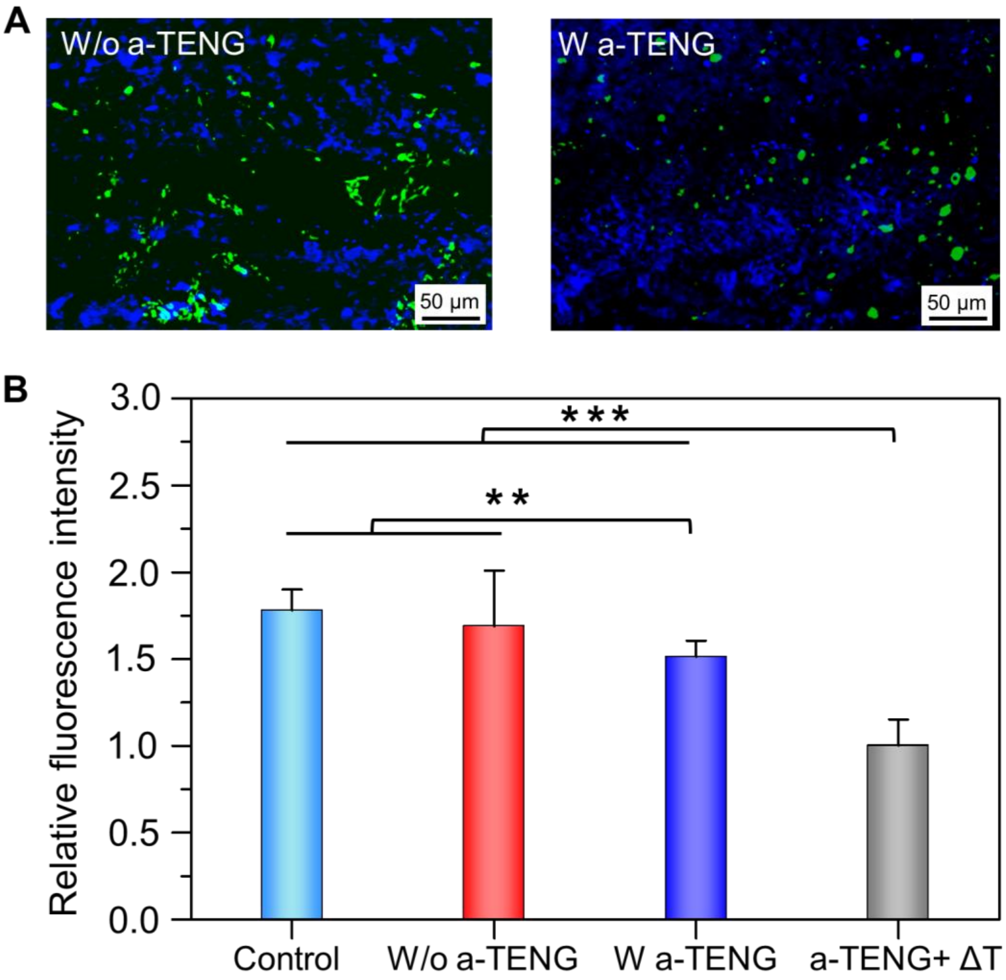

Fig. S25. IL-6 immunofluorescence staining of *S. aureus* infected wound tissues. (A) Images and **(B)** Quantitative analysis of IL-6 expression in wounds on day 9 following different treatments. Results are plotted as mean ± s.d. (n = 6), *$*p < 0.05$, **$p < 0.01$, ***$p < 0.001$* and ****$p < 0.0001$.

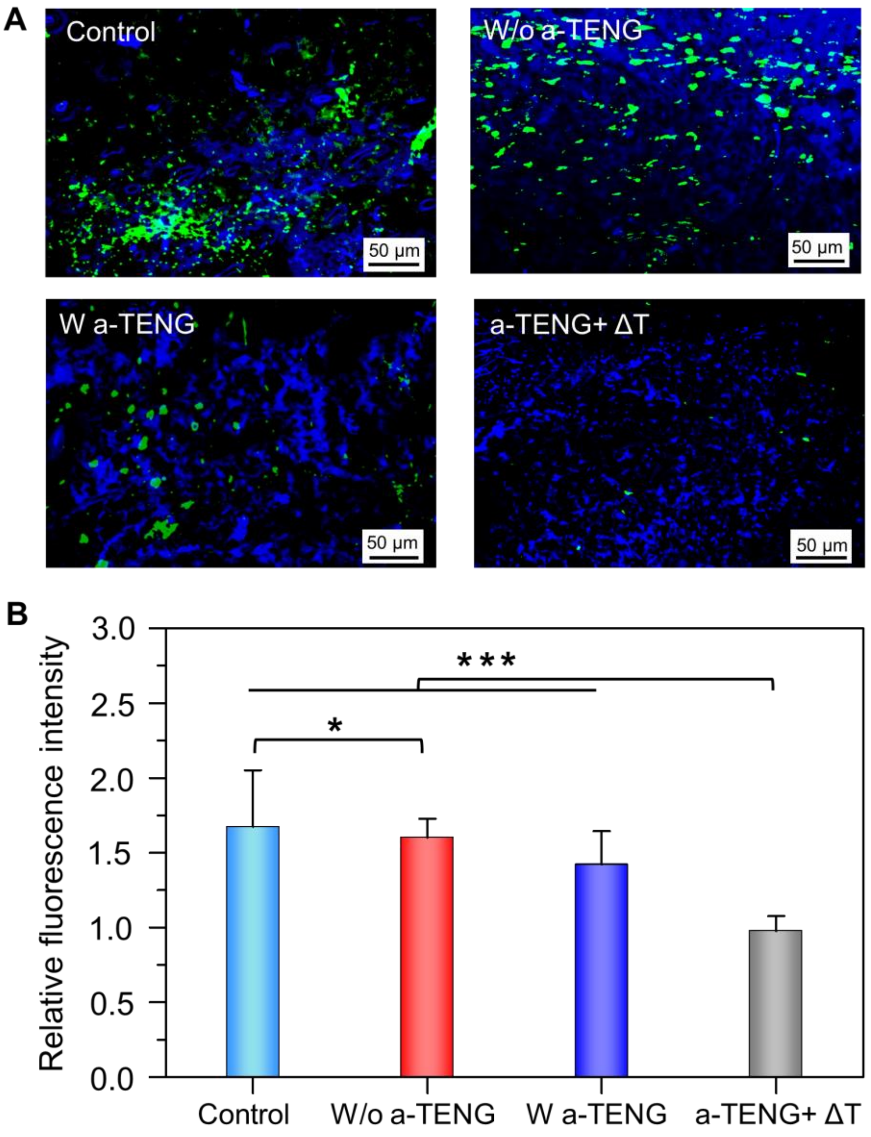

Fig. S26. TNF-α immunofluorescence staining of *S. aureus* infected wound tissues. (A) Images and **(B)** Quantitative analysis of TNF-α expression in wounds on day 9 following different treatments. Results are plotted as mean ± s.d. (n = 6), **p < 0.05, **p < 0.01, ***p < 0.001* and *****p < 0.0001*.

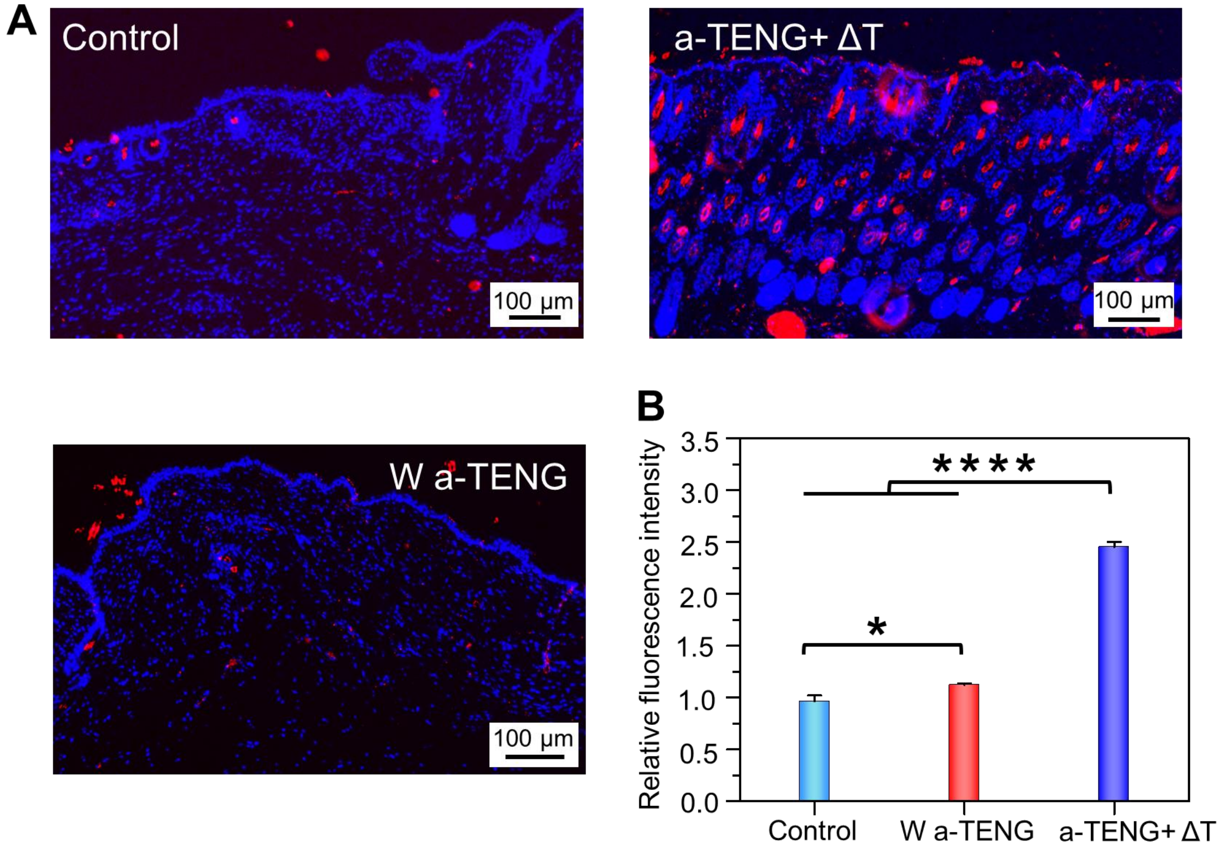

Fig. S27. CD31 immunofluorescence staining of *S. aureus* infected wound tissues. (A) Images and **(B)** Quantitative analysis of CD31 expression in wounds on day 12 following different treatments. Results are plotted as mean ± s.d. (n = 6), $*p < 0.05$, $**p < 0.01$, $***p < 0.001$ and $****p < 0.0001$.

Movie S1.
Output generation from TENG continuously by mice’s motion.
